# Advanced Multifunctional Hydrogels for Enhanced Wound Healing through Ultra‐Fast Selenol‐S_N_Ar Chemistry

**DOI:** 10.1002/advs.202400898

**Published:** 2024-04-22

**Authors:** Yan Wu, Ying Bei, Wenjing Li, Weihong Lu, Jian Zhu, Zhengbiao Zhang, Tinglin Zhang, Sen Liu, Kaiyuan Chen, Hong Jin, Luxin Li, Meng Li, Jie Gao, Xiangqiang Pan

**Affiliations:** ^1^ State and Local Joint Engineering Laboratory for Novel Functional Polymeric Materials Jiangsu Key Laboratory of Advanced Functional Polymer Design and Application College of Chemistry Chemical Engineering and Materials Science Soochow University Suzhou 215123 China; ^2^ College of Life Science Mudanjiang Medical University Mudanjiang 157011 China; ^3^ Changhai Clinical Research Unit Shanghai Changhai Hospital Naval Medical University Shanghai 200433 China; ^4^ Department of Dermatology Shanghai Children's Medical Center School of Medicine Shanghai Jiao Tong University Shanghai 200010 China; ^5^ Shanghai Key Laboratory of Nautical Medicine and Translation of Drugs and Medical Devices Shanghai 200433 China; ^6^ Hainan Academy of Medical Sciences Hainan Medical University Hainan 571199 China

**Keywords:** hydrogel, photoresponsiveness, selenol, S_N_Ar reaction, wound healing

## Abstract

Fabrication of versatile hydrogels in a facile and effective manner represents a pivotal challenge in the field of biomaterials. Herein, a novel strategy is presented for preparing on‐demand degradable hydrogels with multilevel responsiveness. By employing selenol‐dichlorotetrazine nucleophilic aromatic substitution (S_N_Ar) to synthesize hydrogels under mild conditions in a buffer solution, the necessity of additives or posttreatments can be obviated. The nucleophilic and redox reactions between selenol and tetrazine culminate in the formation of three degradable chemical bonds—diselenide, aryl selenide, and dearomatized selenide—in a single, expeditious step. The resultant hydrogel manifests exceptional adaptability to intricate environments in conjunction with self‐healing and on‐demand degradation properties. Furthermore, the resulting material demonstrated light‐triggered antibacterial activity. Animal studies further underscore the potential of integrating metformin into Se‐Tz hydrogels under green light irradiation, as it effectively stimulates angiogenesis and collagen deposition, thereby fostering efficient wound healing. In comparison to previously documented hydrogels, Se‐Tz hydrogels exhibit controlled degradation and drug release, outstanding antibacterial activity, mechanical robustness, and bioactivity, all without the need for costly and intricate preparation procedures. These findings underscore Se‐Tz hydrogels as a safe and effective therapeutic option for diabetic wound dressings.

## Graphical abstract

The nucleophilic and redox reactions between selenol and tetrazine lead to the simultaneous formation of three degradable chemical bonds in a single, rapid step. Consequently, the resulting hydrogel demonstrates exceptional adaptability to intricate environments, self‐healing capabilities, on‐demand degradation potential, as well as light‐triggered antibacterial properties. The preclinical investigations further underscore Se‐Tz hydrogels as a secure and efficacious therapeutic option for diabetic wound dressings.

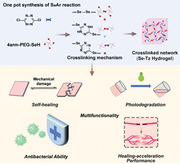

## 1 Introduction

Hydrogels are 3D networks of hydrophilic polymers that can be physically or chemically crosslinked.^[^ ^1^ ^]^ The unique tunable physical properties, intricate structure, and exceptional biocompatibility of these materials enable them to faithfully mimic tissue and cellular microenvironments, rendering them indispensable multifunctional soft materials with diverse applications in the fields of biotechnology and biomedical research, encompassing actuators, sensors, artificial muscles, and tissue engineering, especially wound dressings.^[^ ^1^, ^2^ ^]^ Hydrogels are particularly adept at maintaining local wound temperature and humidity, thus preventing desiccation and subsequent damage to new granulation tissue. This characteristic also serves as a barrier against external bacterial invasion, mitigating nosocomial infections and bolstering local antimicrobial defenses. Considerable efforts have been made by material scientists to develop diverse strategies for the design of hydrogel dressings, exemplified by the utilization of efficient click reactions that enable the construction of functionalized hydrogels with a wide range of applications.^[^ ^3^ ^]^ However, the construction of multifunctional intelligent responsive hydrogel dressings (such as controlled administration, antibacterial property, and on‐demand degradation) remains an arduous challenge, necessitating the development of simple yet effective methodologies to address this issue.^[^ ^4^ ^]^

Advanced hydrogel dressings for biomedical applications often require precise regulation and intelligent responses under mild conditions. Photoresponsive hydrogels are favorable for achieving actively controlled responses by regulating the intensity and wavelength of light, as well as the duration of illumination.^[^ ^5^ ^]^ Despite the development of a diverse range of photoresponsive hydrogels by material chemists, there is significant room for optimization in terms of intricate design and synthesis processes, limited light wavelength coverage, inadequate penetration capabilities, and insufficient biocompatibility.^[^ ^6^ ^]^ Achieving precise spatiotemporal control of drug release remains a formidable challenge due to the inherent difficulty in modulating the release process at will. Therefore, it is imperative to rationally design hydrogels with precise spatiotemporal response properties.^[^ ^7^ ^]^ The photoresponsiveness of hydrogels is typically achieved by incorporating functional groups into the system rather than relying on the intrinsic properties of the polymer or supramolecular structure itself.^[^ ^5^, ^8^ ^]^ Various photochromic or photocleavage groups, such as azobenzene,^[^ ^9^ ^]^ spiropyran,^[^ ^10^ ^]^ *o*‐nitrobenzyl,^[^ ^11^ ^]^ and coumarin,^[^ ^12^ ^]^ are commonly introduced into the structure of responsive hydrogels. Upon irradiation, these photoresponsive chromophores can induce reversible or irreversible gel‐sol transitions and regulate the release of encapsulated drugs.^[^ ^13^ ^]^ To overcome the limitations associated with high‐energy ultraviolet (UV) light and poor light penetration, special reagents are sometimes added to enable a near‐infrared light response.^[^ ^14^ ^]^ Despite significant efforts in designing and investigating photoresponsive hydrogels, most of the existing systems are limited to proof‐of‐concept studies. Moreover, these gel systems often require complex experimental setups involving intricate synthesis and purification processes. To facilitate practical applications of larger‐scale photoresponsive hydrogels, several key challenges need to be addressed. First, linkers should exhibit enhanced responsiveness to mild light to avoid reliance on high‐energy light with limited penetration while minimizing phototoxicity toward normal cells and tissues. Second, simpler and more efficient reaction systems should be developed for constructing multistage response systems and eliminating complex steps so that hydrogels can be widely utilized. Green light (GL, 520–560 nm) offers a promising alternative, with its capacity for deeper tissue penetration and minimal invasiveness. The advancement of GL‐responsive materials, particularly the discovery of appropriate photocleavable groups, is pivotal for the engineering of hydrogels that can be effectively cleaved under GL irradiation. The swift kinetics and high efficiency of GL‐induced photochemical reactions hold particular promise for their application in medicine. Furthermore, empirical data indicate that GL irradiation can accelerate the wound healing process and possess anti‐inflammatory properties.^[^ ^15^ ^]^

**Scheme 1:**
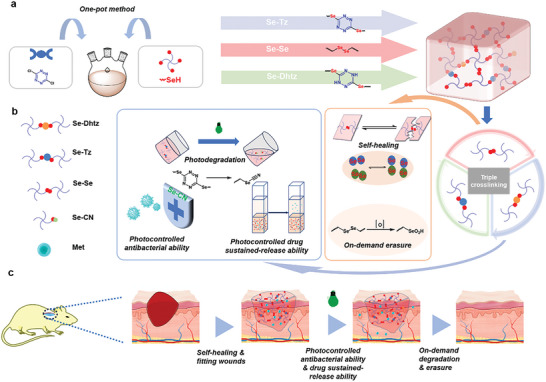
The Se‐Tz hydrogel was synthesized using a one‐pot, cost‐effective, and environmentally friendly approach by combining selenol with tetrazine. The hydrogel possesses three chemical cross‐linking mechanisms (diselenide, aryl selenide, and dearomatized selenide bonds), endowing it with unique properties, such as photodegradation, photocontrolled release, on‐demand dissolution, and self‐healing capabilities. Additionally, animal studies have demonstrated the light‐triggered antibacterial activities of hydrogels.

Here, we present a novel approach for the one‐pot fabrication of multilevel spatiotemporally responsive hydrogels using selenol‐dichlorotetrazine nucleophilic aromatic substitution (Se‐S_N_Ar) chemistry. Tetrazine undergoes both nucleophilic and reductive reactions with selenol, resulting in the formation of three degradable chemical bonds: diselenide (Se‐Se), aryl selenide (Se‐Tz), and dearomatized selenide bonds (Se‐Dhtz). This one‐step ultrafast synthesis allows for the incorporation of three cross‐linking points, each corresponding to a specific response condition. Tetrazine is a versatile structural unit commonly used in the construction of biomedical materials, including bioorthogonal reactive and photodegradable materials.^[^ ^16^ ^]^ However, numerous studies have focused on the click chemistry efficiency of tetrazine and its application in functionalized labeling, with limited attention given to its utilization in the development of smart hydrogels that achieve multiple responses and exhibit active functionalities.^[^ ^17^ ^]^ In this study, we successfully synthesized a Se‐Tz hydrogel, which can be triggered by gentle 520 nm light to form selenocyanate (SeCN)‐terminated PEG. This novel hydrogel exhibits antibacterial and anti‐inflammatory activities comparable to those of small‐molecule selenocyanate compounds.^[^ ^18^ ^]^ The presence of diselenide in the system enables dynamic exchange under visible light irradiation, allowing the hydrogel to adapt to various states. It maintains its form and ensures continuous photoresponsive drug release while firmly adhering to the wound surface, ensuring the stability of the material shape. Finally, the hydrogel can be removed on demand by the addition of 0.03% hydrogen peroxide, which completely breaks both diselenide bonds and Se‐Dhtz bonds, resulting in dissolution and erasure. Furthermore, programmed regulation of the hydrogel's structure enables precise release of encapsulated metformin,^[^ ^19^ ^]^ promotes angiogenesis, and further promotes wound healing in the diabetic bed. Importantly, these hydrogels can be prepared without costly or complicated procedures, making them safe and efficient therapeutic options for diabetic wound dressings (**Scheme** **1**).

## 2 Results and Discussion

### 2.1 Model Reaction of Selenol and Dichloromethyltetrazine

**Figure 1:**
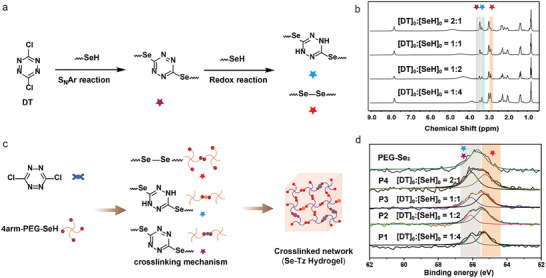
a) Mechanism of the reaction between selenol and DT; b) ^1^H NMR spectra of selenol reacting with DT in varying proportions; c) Fabrication of model triple cross‐linked hydrogels through S_N_Ar substitution and the redox reaction of selenol‐dichlorotetrazine; d) XPS spectra of Se 3d resulting from the reaction of selenol with dichloromethane in different proportions.

It is well established that dichlorotetrazine can undergo reactions with various nucleophiles, including alcohols, amines, thiols, and others.^[^ ^20^ ^]^ However, there are a lack of reports in the literature regarding the nucleophilic substitution of selenols for dichlorotetrazine (DT), and we present here the first use of the aryl nucleophilic reaction of selenol to synthesize tetrazinyl selenide. The nucleophilicity of selenol is exceptionally high, leading to an ultrafast reaction rate. Further examination of the ^1^H nuclear magnetic resonance (NMR) and liquid chromatography‐mass spectrometry (LC‐MS) analysis after the addition of selenol to DT revealed rapid completion of the reaction (Figure S1, Supporting Information), surpassing even the reactivity observed in thiol‐based nucleophilic reactions.^[^ ^21^ ^]^ This enhanced reactivity can be attributed to both the larger atomic radius of the selenium atoms and the strong acidity of the selenols. With a *p*Ka as low as 5, they readily delocalize to generate Se negative ions and exhibit potent nucleophilic ability.^[^ ^22^ ^]^ By employing ^1^H NMR and LC‐MS characterization techniques (Figure S2, Supporting Information), we discovered not only tetrazinyl selenide but also diselenide and dihydrotetrazinyl selenide as products (**Figure** **1**a). This occurrence arises from the powerful reducing capability of selenol molecules, which can dearomatize tetrazines while simultaneously oxidizing themselves into diselenide compounds.^[^ ^22^ ^]^ Upon the addition of excess selenol due to its strong reductivity (Figure S2b, Supporting Information), seleno‐tetrazine is reduced to seleno‐dihydrotetrazine—a rare observation in aryl nucleophilic reactions—whereas hydroxyl or amino‐based nucleophilic reactions do not exhibit such reduction phenomena. Although sulfhydryl‐based nucleophilic reactions were found to yield reduction byproducts,^[^ ^23^ ^]^ their quantities were minimal, and there are no reported means for regulating or optimizing these reactions.^[^ ^24^ ^]^ Therefore, the reaction between selenol and DT provides an opportunity for the synthesis of multifunctional linkers in a single step, wherein the composition of these linkers can be tailored by adjusting the [SeH]/[DT] ratio (as confirmed by ^1^H NMR analyses, Figure 1b).

According to the properties of tetrazines,^[^ ^20^, ^23^ ^]^ the structure of tetrazinyl selenide exhibits potential photoresponsive degradation performance (Figure S3a, Supporting Information). The photoresponse properties of the small molecule tetrazine selenide were investigated under green light (520 nm) irradiation. The absorption peak intensity gradually decreased over time and reached equilibrium after 800 min (Figure S3a,b, Supporting Information). NMR, gas chromatography‐time of fight‐mass spectrometry (GCT‐TOF‐MS) and fourier transform infrared spectroscopy (FT‐IR) analysis of the reaction solution (Figure S3c–e, Supporting Information) revealed that the decomposition product corresponded to a selenocyanide compound, consistent with the photolysis mechanism of tetrazinyl sulfide.^[^ ^17^, ^20^ ^]^ Upon light excitation, a single tetrazine molecule decomposes to yield one nitrogen molecule and two selenocyanate (SeCN) molecules, and these compounds have been experimentally proved to have significant biological activities., such as antioxidant and antibacterial effects.^[^ ^25^ ^]^

### 2.2 Preparation and Characterization of the Se‐Tz Hydrogel

**Figure 2:**
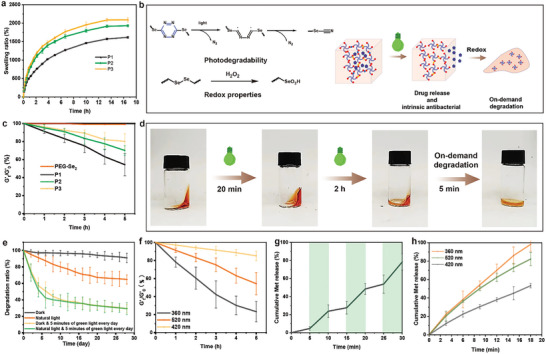
Performance testing of hydrogels. a) Swelling kinetics of hydrogels in PBS at pH 7.4 and 37 °C. b) Degradation mechanism and schematic diagram of the hydrogel. c) Relationships between the ratios of the storage modulus (G′_t_) of different hydrogels and the initial storage modulus (G″_0_) over time under 520 nm green light (10 W cm^−2^). d) The sequential stages of degradation through photographic documentation. e) The change in weight loss rate with time during degradation of the P1 hydrogel in vitro under different conditions. f) Relation between the ratio of the storage modulus (G′_t_) of P1 to the initial storage modulus (G″_0_) with time under different light conditions. g) Drug release curve of the Se‐Tz hydrogel loaded with metformin under intermittent 520 nm green light irradiation. h) The drug release profile of the Se‐Tz hydrogel loaded with metformin was exposed to light sources of varying wavelengths for a duration of 10 min. The data are presented as the means ± SDs (*n* = 3).

Subsequently, a hydrogel was developed based on the reaction between four‐arm polyethylene glycol (PEG) selenol and DT, as illustrated in Figure 1c. The reaction between selenol and DT was observed to exhibit remarkable kinetics, leading to instantaneous gel formation in a pH 7.4 buffer solution regardless of whether selenol was introduced into the DT solution or vice versa (Video S1, Supporting Information). Four distinct types of hydrogels were synthesized by adjusting the molar ratio of SeH/DT functional groups and subsequently characterized using X‐ray photoelectron spectroscopy (XPS) and FT‐IR techniques (Figure 1d; Figure S4c, Supporting Information). XPS analysis revealed that an increase in the proportion of selenol led to a greater degree of reduction and an elevated content of diselenide. Notably, no significant decrease was observed in the monoselenide region encompassing Se‐Tz or Se‐Dhtz. Due to the excessive presence of monosubstituted tetrazine in the polymer structure (P4) at a 2:1 ratio, the gelation ability of the reaction system decreased significantly, thus limiting further discussion on this matter within the scope of this paper (Figure S4, Supporting Information). Swelling kinetics demonstrated that equilibrium could be achieved for all three hydrogels (P1‐P3), with equilibrium swelling rates reaching 1600%, 1900%, and 2000%, respectively (**Figure** **2**a). According to our previous investigation, the dynamic exchange capability of diselenide can induce unbounded expansion of the hydrogel until complete “dissolution” occurs.^[^ ^22^ ^]^ This observation suggested that nondynamic covalent bonding of dihydrotetrazine occurs within these systems, effectively preventing indefinite expansion of the hydrogels. Therefore, we engineered the gel system to rely on dihydrotetrazine bonds to maintain its structural integrity. In addition, the hydrogel's superior swelling properties ensure maintenance of a moist environment at the wound site, crucial for healing, while concurrently absorbing substantial wound exudate. The self‐healing property of the hydrogel was achieved through dynamic diselenide exchange. Additionally, the photosensitivity of selenotetrazine provided exceptional photocontrolled degradation as well as antioxidant properties in the hydrogel.

To assess the mechanical properties of the hydrogels, a series of rheometer tests were conducted. The results from time scanning and strain scanning for polymers P1‐P3 (Figure S5, Supporting Information) indicate that the storage modulus of all three polymers exceeds the loss modulus, confirming their classification as hydrogels. Furthermore, these hydrogels exhibit stability under low‐strain conditions. The hydrogels’ energy storage modulus (G′) ranges from 1000–3000 Pa, with a loss modulus (G″) ≈10% of the former, mirroring the viscoelastic properties of the extracellular matrix and thus promoting cellular growth and proliferation.^[^ ^26^ ^]^ The frequency scanning results for polymers P1‐P3 (Figure S6, Supporting Information) demonstrated shear thinning, highlighting their remarkable injectability. By subjecting the hydrogels to 50 loading‒unloading rotating test cycles with a constant strain of 100%, their recovery and resilience could be assessed (Figure S7, Supporting Information). The experimental findings revealed exceptional fatigue resistance in these hydrogels, with 50 cycles of stress‐to‐initial stress ranging from 98.62% to 71.46%. Specifically, an increased concentration of dynamic covalent diselenide enhances fatigue resistance while increasing the content of Se‐Dhtz. decreases it. Rheological recovery was evaluated through continuous step strain testing (Figure S8, Supporting Information). After experiencing an initial high strain of 3000%, there was a significant decrease in G′, indicating a collapse within the hydrogel network; moreover, G″ surpassed G′. In the presence of a low strain (1%), the G′ of the hydrogels rebounded, implying the restoration of cross‐linking. Therefore, there is a positive correlation between the diselenide content and both the fatigue resistance and self‐healing ability of hydrogels.

The presence of Se‐Tz bonds in the hydrogel can lead to structural degradation under irradiation, as evidenced by a significant decrease in the storage modulus G′. Moreover, this decrease was more pronounced with increasing Se‐Tz content (Figure 2c). To be specific, the photodegradation mechanism of tetrazine involves a photochemical reaction where the tetrazine molecule absorbs light and undergoes structural changes. The intermediate can then undergo further reactions, such as rearrangement, to form the final degradation products — nitrogen and selenocyanate (Figure 2b).^[^ ^27^ ^]^ This degradation process was monitored using UV‐vis absorption spectroscopy (Figure S9a,b, Supporting Information). The absorption of the compounds gradually decreased within the range of 400 to 500 nm and reached equilibrium after 160 min. Consequently, we are able to precisely control the photoinduced degradation of the hydrogel. In the absence of irradiation, the hydrogel exhibited remarkable stability over a period of 28 days, with only a marginal loss of hydrogel mass amounting to 9%. This slight loss may be attributed to dynamic diselenide exchange within the system.^[^ ^28^ ^]^ Under exposure to natural light (laboratory conditions), ≈35% of the hydrogel mass was lost after 28 days; however, when subjected to green light for just 5 min per day, rapid degradation occurred, with an observed decrease of up to 48% within 5 days. As diselenide also responds sensitively to green light stimulation, the total mass loss could eventually reach as high as 70% (Figure 2e). After reaching equilibrium on day 28, further photodegradation was restricted by the presence of nonphotoresponsive tetrahydroselenide bonds in the system, resulting in <30% of the hydrogel remaining. The hydrogel also exhibited the capability to modulate photodegradation through the utilization of light at various wavelengths. By subjecting the hydrogel to continuous irradiation at different wavelengths, controlled degradation can be achieved at distinct time scales (Figure 2f). After 5 h of continuous UV light (365 nm) irradiation, the G′_t_/G′_0_ ratio can be reduced to 23% as a result of the excitation of weak diselenide and Se‐Tz bonds by high‐energy light. Under 520 nm light irradiation, the G′_t_/G′_0_ ratio decreased to 54%, while under 420 nm light irradiation, it could only be reduced to 85% since only diselenide was activated and degradation of the Se–Tz bond did not occur. The hydrogel exhibited continuous softening under sustained illumination while still maintaining a distinct morphology attributed to the presence of diselenide and dihydrotetrazine moieties, which could be completely degraded upon the addition of H_2_O_2_ (0.3%) (Figure 2d). These findings demonstrated that the degradation performance of our designed hydrogel has multiple control properties and can be erased on demand. Furthermore, we can adjust the degradation rate by manipulating both the power and wavelength of the light (Figure 2f; Figure S9c, Supporting Information). This ensures that in practical applications, milder light with better tissue penetration capabilities can be utilized without causing harm. When a model drug such as metformin hydrochloride is incorporated into our system, controlled release is achievable through precise control of the illumination wavelength and power (Figure 2g,h; Figure S9d, Supporting Information).

### 2.3 In Vivo and In Vitro Antibacterial Activity of Se‐Tz

**Figure 3:**
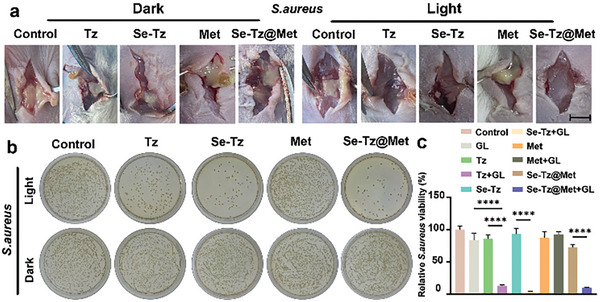
Antibacterial activity of the hydrogels and Tz. a) Images of the wounds after different treatments at different time points, bar represent 5 mm. b) Images of plates showing the bacterial colony formation assay in the presence or absence of green light irradiation. c) Relative viability of bacterial colonies calculated from b). ^*^ *p* < 0.05; ^**^ *p* < 0.01; ^***^ *p* < 0.001; ^****^ *p* < 0.0001. The data are presented as the means ± standard deviations (*n* = 3).

Selenocyanates have been extensively investigated and applied in the medical field due to their wide range of antibacterial, anti‐inflammatory, and antioxidant activities.^[^ ^24^ ^]^ These compounds effectively impede bacterial and fungal growth, thus demonstrating significant potential as promising therapeutic agents for combating infectious diseases. In light of these findings, we postulated that polymer modifications incorporating selenocyanate groups would result in similar activities. To explore this hypothesis, we investigated the photodegradation of Se–Tz bonds in a hydrogel, which led to the formation of SeCN groups known for their potential antibacterial properties. Subsequently, antibacterial activity assays were conducted using a small molecule (Tz), a Se‐Tz hydrogel (Se‐Tz), a metformin (Met) and a hydrogel containing metformin (Se‐Tz@Met). Colony formation assays were performed on spread plates to evaluate the antibacterial capacities of Tz, Se‐Tz, and Se‐Tz@Met (**Figure** **3**; Figure S10, Supporting Information). Under conditions without green light, the strains exhibited weak antibacterial ability against *Staphylococcus aureus* (*S. aureus*). However, following 15 min of exposure to 520‐nm green light irradiation, the Tz, Se‐Tz, and Se‐Tz@Met groups demonstrated significantly reduced relative viability of *S. aureus*, with viability percentages of 12% ± 0.4%, 3% ± 1%, and 4% ± 0.2%, respectively (Figure S10a,b, Supporting Information). These findings unequivocally demonstrate the potent antibacterial activity of Tz, Se‐Tz, and Se‐Tz@Met against *S. aureus* under green light irradiation.

The therapeutic effectiveness of Tz, Se‐Tz, and Se‐Tz@Met was further assessed using an in vivo wound infection model caused by *S. aureus* in mice. Following the inoculation of a bacterial solution, the wounds were subjected to various posttreatment care methods. On the 7th day, the wound tissue was examined and photographed. Notably, the Tz, Se‐Tz, and Se‐Tz@Met groups exposed to 520 nm green light exhibited significant disinfection effects without any visible signs of infection, while evident infection with visible pus formation was observed in the other groups at the wound site (Figure 3a). Bacterial counts in *S. aureus*‐infected wounds were evaluated using a plate colony formation assay. No significant differences were observed among the groups without light irradiation regarding *S. aureus* counts. However, upon light irradiation treatment, the Tz, Se‐Tz, and Se‐Tz@Met groups demonstrated significantly lower relative viabilities than did the control group, consistent with their superior antibacterial activity observed in vitro (Figure 3b,c).

Furthermore, we evaluated the antibacterial activity of Tz, Se‐Tz, and Se‐Tz@Met against *Escherichia coli* (*E. coli*) and Methicillin‐resistant *Staphylococcus aureus* (MRSA) in vitro by a plate colony formation assay. The results clearly demonstrated that Tz, Se‐Tz, and Se‐Tz@Met effectively combat both *E. coli* and MRSA when exposed to green light irradiation (Figure S10c–f, Supporting Information). Overall, these results demonstrate that under 520 nm light irradiation, the Tz, Se‐Tz, and Se‐Tz@Met groups exhibit superior antibacterial performance.

The application of antibacterial hydrogels as effective and versatile tools holds significant promise for a wide array of clinical uses.^[^ ^28^, ^29^ ^]^ However, potential limitations associated with these hydrogels, including their potential to elicit toxic or allergenic responses in human tissues, contribute to bacterial resistance, and have adverse environmental effects, should be considered.^[^ ^30^ ^]^ Prolonged exposure to subtherapeutic concentrations of antibiotics in these hydrogels has been linked to the development of antibiotic resistance.^[^ ^31^ ^]^ In this study, our Tz, Se‐Tz, and Se‐Tz@Met hydrogels demonstrated robust antibacterial activity against *E. coli* and MRSA exclusively in the presence of green light irradiation, thereby minimizing prolonged contact between antibacterial agents and bacteria. The concept of “photoelicited antibacterial activity” offers a potential overarching strategy to address antibiotic resistance.

### 2.4 Proliferation and Migration Efficacy of the Hydrogels and Met

The biocompatibilities of Tz, Se‐Tz, Met, and Se‐Tz@Met were evaluated using NIH‐3T3 fibroblasts and Human Umbilical Vein Endothelial Cells （HUVECs（. Calcein‐AM/PI staining did not reveal any significant decrease in cellular viability or alterations in cellular morphology among cells treated with Tz, Se‐Tz, Met, or Se‐Tz@Met (Figure S11a,b, Supporting Information). These results indicated that Tz, Met, and the hydrogels had minimal effects on cell viability and did not induce cellular damage, regardless of exposure to green light, demonstrating their compatibility with cell growth.

To investigate the potential effects of released Met on NIH‐3T3 cell and HUVEC behavior in vitro, we assessed cell proliferation and migration after coculturing with Se‐Tz@Met hydrogels. MTT assays were performed to evaluate NIH‐3T3 cell and HUVEC proliferation after 24 h of culture (Figure S11c,d, Supporting Information). The cell viability in the Tz group remained consistent at ≈100% within the concentration range of 0–50 µg mL^−1^, irrespective of light exposure, suggesting that Tz did not possess any proproliferative properties. The Met group exhibited a proproliferative effect within the concentration range of 0–50 µg mL^−1^, regardless of light exposure. Specifically, the proliferation rate of NIH‐3T3 cells in the Met+GL group reached 49% ± 5.3% at a concentration of 50 µg mL^−1^, while the value‐added rate of HUVECs was 58% ± 2.6%. Following Se‐Tz hydrogel treatment, a notable increase in cell viability was observed, particularly in the light group, when the cells were exposed to 500 µg mL^−1^ Se‐Tz. The Se‐Tz@Met group demonstrated a significant increase in cell count following a 24 h treatment period, and the presence of light further augmented this effect. Similarly to those in the Met group, the proliferation rate of NIH‐3T3 cells in the Se‐Tz@Met group at a concentration of 500 µg mL^−1^ was 42% ± 4.5%, and that of HUVECs was 52% ± 5.6%. These results indicate that the sustained release of Met promoted NIH‐3T3 cell and HUVEC proliferation.

Furthermore, the cell migration assay results (Figure S12a,c, Supporting Information) confirmed that, compared with the control, Se‐Tz, Met, and Se‐Tz@Met significantly enhanced the motility of NIH‐3T3 cells and HUVECs. Additionally, the number of migrating cells was significantly greater in the Se‐Tz@Met+GL group than in the Se‐Tz@Met group for both NIH‐3T3 cells and HUVECs (Figure S12b,d, Supporting Information). These findings suggest that the Se‐Tz hydrogels exhibited excellent biocompatibility and that the sustained release of Met from the hydrogels effectively activated a series of beneficial behaviors, including cell proliferation and migration, in NIH‐3T3 cells and HUVECs in vitro.

### 2.5 In Vivo Wound Healing in a Diabetic Mouse Model

The in vitro experiments provided evidence that the Se‐Tz hydrogel has potential as a viable option for chronic diabetic wound dressings. Subsequently, a diabetic mouse model was utilized to evaluate the efficacy of the hydrogels in wound healing. ICR mice were intraperitoneally administered streptozotocin (STZ), followed by the induction of a full‐thickness wound with a diameter of 6 mm in mice displaying persistent blood glucose levels surpassing 16.7 mmol L^−1^.

**Figure 4:**
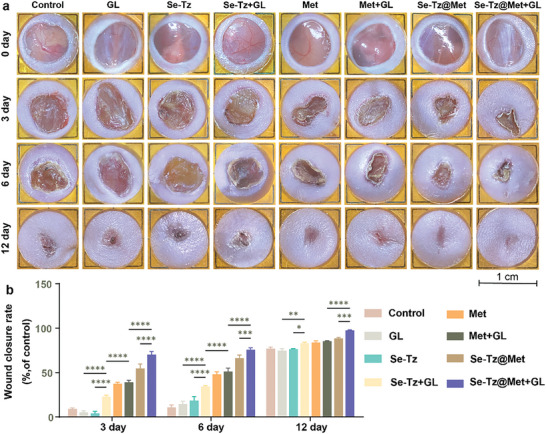
Wound healing of diabetic wounds promoted by hydrogels and Met. a) Representative images depicting the wound healing process in mice treated with different interventions at different time points. Bar represent 1 cm. b) Wound closure rates in mice treated with different interventions at different time points. ^*^ *p* < 0.05; ^**^ *p* < 0.01; ^***^ *p* < 0.00; ^****^ *p* < 0.0001. The data are presented as the means ± SDs (n = 3).

The therapeutic efficacy of the various regimens was subsequently assessed on days 0, 3, 6, and 12 in the different groups, namely, the control group, GL group, Se‐Tz group, Se‐Tz+GL group, Met group, Met+GL group, Se‐Tz@Met group, and Se‐Tz@Met+GL group. Macroscopically, as shown in **Figure** 4a,b, the Se‐Tz+GL, Met, Met+GL, Se‐Tz@Met, and Se‐Tz@Met+GL groups exhibited faster wound healing than the control group. The Se‐Tz@Met+GL group demonstrated the highest healing ratio (98% ± 0.2%) on day 12, while the Se‐Tz@Met group achieved a healing rate of only 88% ± 1% (*P* < 0.05), indicating that Se‐Tz@Met and green light irradiation synergistically accelerated wound healing. Taken together, the results demonstrated that, compared with the other groups, the Se‐Tz@Met + GL group exhibited superior wound healing ability.

### 2.6 Histomorphological Evaluation

Histological examination was also conducted to evaluate the quality of the regenerated skin in diabetic wounds (Figure S13, Supporting Information). Tissue samples from each group of mice were stained with Hematoxylin and eosin (H&E) and Masson's trichrome. The assessment of wound length on the sixth day revealed that, compared to those in the control group (5.39 mm ± 0.1 mm), all the treatment groups, except for the Se‐Tz group, had shorter wounds. In particular, the Se‐Tz@Met + GL group exhibited the most significant reduction in wound length, measuring 2.74 mm ± 0.3 mm (*P* < 0.05). Additionally, except for those in the Se‐DT group, all the treatment groups had shorter wounds on the twelfth day than did the control group (3.08 mm ± 0.1 mm), with the Se‐Tz@Met + GL group showing the most minimal difference in wound length (*p* < 0.05) (Figure S13a,d, Supporting Information).

Granulation tissue plays a vital role in such wounds by initially proliferating, degrading and absorbing foreign bodies and necrotic tissues to fill the wound, and finally transforming into scar tissue to seal it. Therefore, the progression of more compact granulation tissue during the course of wound healing is a crucial determinant of effective wound healing. As shown in Figure S13b,e (Supporting Information), on day 6, the control group exhibited less dense granulation tissue than the other treatment groups did, excluding the Se‐Tz group (*p* < 0.05). Notably, the Se‐Tz@Met + GL group displayed the most substantial increase in tissue thickness. These H&E staining results suggested that the wounds treated with Se‐Tz@Met + GL achieved relatively satisfactory healing, as indicated by not only a fast‐healing rate but also skin appendages in the scar area.

As healing progresses, granulation tissue is gradually replaced by collagen fibers. The initial fibrin‐fibronectin clot that forms during the early stages of wound healing is progressively substituted by collagen, leading to increased wound strength and resilience against subsequent damage. Cells involved in angiogenesis and connective tissue development utilize collagen as a scaffold for adhesion, proliferation, and differentiation. Figure S13a,c,f (Supporting Information) demonstrates the deposition of newly synthesized collagen in the wounds. On day 12, the control group, the GL group, and the Se‐Tz group exhibited sparse and disorganized low‐level collagen deposition, whereas the other treatment groups, especially the Se‐Tz@Met + GL group, exhibited significantly increased collagen deposition compared to the control group, the GL group, and the Se‐Tz group. Moreover, skin appendages were also prominently observed in the Se‐Tz@Met + GL group, confirming the results of the above H&E staining. Wounds treated with Se‐Tz@Met + GL exhibited satisfactory healing, as evidenced by H&E and Masson staining, with a rapid healing rate and collagen synthesis at the wound site.

Immunohistochemical staining of CD206 (anti‐inflammatory factors), CD86 (pro‐inflammatory factors), and Ki67 (a marker of cell proliferation) was performed on wound tissue to investigate the mechanism by which the prepared hydrogel promotes wound healing in diabetic mice. As shown in Figure S14a–d (Supporting Information), the Se‐Tz@Met+GL group exhibited the least intense positive staining of CD86, along with elevated levels of CD206 in a diabetic mice wound model on days 6. These findings suggested that Se‐Tz@Met+GL demonstrated superior in vivo anti‐inflammatory properties and effectively inhibited the release of proinflammatory cytokines in diabetic wounds. Additionally, Ki67 expression was also assessed in wound samples on days 12. The Se‐Tz@Met+GL group exhibited the highest statistically significant expression of the Ki67 marker compared to the other groups (Figure S14e,f, Supporting Information). In conclusion, the Se‐Tz@Met+GL group facilitated cell proliferation and suppressed inflammation during the healing process, resulting in accelerated and improved healing of diabetic wounds.

## 3 Conclusion

In this study, we introduce a novel design for an on‐demand degradable hydrogel with a multilevel response. The hydrogel is synthesized through nucleophilic aromatic substitution of selenol‐dichlorotetrazine under mild conditions in a buffer solution, eliminating the need for additives or posttreatment. The nucleophilic and redox reactions between selenol and tetrazine lead to the formation of three degradable chemical bonds—diselenide, aryl selenide, and dearomatized selenide—in a single, ultrafast step. The resulting hydrogel exhibited exceptional adaptability to complex environments, as did self‐healing and on‐demand degradation properties. Additionally, it has light‐triggered antibacterial effects. Animal studies underscore the potential of incorporating metformin into Se‐Tz hydrogels under green light irradiation, as it effectively promotes angiogenesis and collagen deposition, thereby facilitating efficient wound healing. In comparison to previously reported hydrogels, Se‐Tz hydrogels exhibit controlled degradation and drug release and outstanding antibacterial activity, mechanical strength, and bioactivity, all without the need for costly and intricate preparation procedures. These findings emphasize the use of Se‐Tz hydrogels as a safe and efficient therapeutic option for diabetic wound dressings.

## Conflict of Interest

The authors declare no conflict of interest.

## Supporting information

Supporting Information

Supplemental Video 1

## Data Availability

The data that support the findings of this study are available in the supplementary material of this article.

## References

[1] a) J. Li , D. J. Mooney , Nat. Rev. Mater. 2016, 1, 16071; 29657852 10.1038/natrevmats.2016.71 PMC5898614

[2] a) S. Li , L. Wang , W. Zheng , G. Yang , X. Jiang , Adv. Funct. Mater. 2020, 30, 2002370;

[3] a) Y. Ren , J. Guo , Z. Liu , Z. Sun , Y. Wu , L. Liu , F. Yan , Sci. Adv. 5, eaax0648; 31467977 10.1126/sciadv.aax0648 PMC6707778

[4] a) M. Vázquez‐González , I. Willner , Angew. Chem., Int. Ed. 2020, 59, 15342; 10.1002/anie.201907670 31730715

[5] a) K. Kalayci , H. Frisch , V. X. Truong , C. Barner‐Kowollik , Nat. Commun. 2020, 11, 4193; 32826921 10.1038/s41467-020-18057-9 PMC7443129

[6] a) P. Lavrador , M. R. Esteves , V. M. Gaspar , J. F. Mano , Adv. Funct. Mater. 2021, 31, 2005941;

[7] H. Zhu , H. Yang , Y. Ma , T. J. Lu , F. Xu , G. M. Genin , M. Lin , Adv. Funct. Mater. 2020, 30, 2000639. 32802013 10.1002/adfm.202000639 PMC7418561

[8] R. Raman , T. Hua , D. Gwynne , J. Collins , S. Tamang , J. Zhou , T. Esfandiary , V. Soares , S. Pajovic , A. Hayward , R. Langer , G. Traverso , Sci. Adv. 6, eaay0065. 32010768 10.1126/sciadv.aay0065 PMC6968934

[9] a) Z. L. Pianowski , J. Karcher , K. Schneider , Chem. Commun. 2016, 52, 3143; 10.1039/c5cc09633b 26804160

[10] a) M. Bril , A. Saberi , I. Jorba , M. C. van Turnhout , C. M. Sahlgren , C. V. C. Bouten , A. P. H. J. Schenning , N. A. Kurniawan , Adv. Sci. 2023, 2303136; 10.1002/advs.202303136 PMC10625123 37740666

[11] a) K. M. C. Tsang , N. Annabi , F. Ercole , K. Zhou , D. J. Karst , F. Li , J. M. Haynes , R. A. Evans , H. Thissen , A. Khademhosseini , J. S. Forsythe , Adv. Funct. Mater. 2015, 25, 977; 26327819 10.1002/adfm.201403124 PMC4551408

[12] a) M. Claudino , X. Zhang , M. D. Alim , M. Podgórski , C. N. Bowman , Macromolecules 2016, 49, 8061; 28989189 10.1021/acs.macromol.6b01605 PMC5630186

[13] a) T. L. Rapp , C. A. DeForest , Nat. Commun. 2023, 14, 5250; 37640707 10.1038/s41467-023-40805-w PMC10462736

[14] a) S. Wu , H.‐J. Butt , Adv. Mater. 2016, 28, 1208; 26389516 10.1002/adma.201502843

[15] a) P. Dungel , S. Sutalo , C. Slezak , C. Keibl , B. Schädl , H. Schnidar , M. Metzger , B. Meixner , J. Hartmann , J. Oesterreicher , H. Redl , P. Slezak , Int. J. Mol. Sci. 2023, 24, 5895; 36982967 10.3390/ijms24065895 PMC10054229

[16] a) A. M. Tallon , Y. Xu , G. M. West , C. W. am Ende , J. M. Fox , J. Am. Chem. Soc. 2023, 145, 16069; 37450839 10.1021/jacs.3c04444 PMC10530612

[17] a) C. Wang , C. Liu , Q. Wei , L. Yang , P. Yang , Y. Li , Y. Cheng , Research 2020, 2020, 6563091; 33015634 10.34133/2020/6563091 PMC7510344

[18] a) M. Sarfraz , M. J. Nasim , M. C. H. Gruhlke , J. Handzlik , C. Jacob , Antibiotics 2023, 12, 290; 36830201 10.3390/antibiotics12020290 PMC9952309

[19] a) A. Caturano , R. Galiero , P. C. Pafundi , JAMA, J. Am. Med. Assoc. 2019, 322, 1312; 10.1001/jama.2019.11489 31573630

[20] a) T. Santos , D. S. Rivero , Y. Pérez‐Pérez , E. Martín‐Encinas , J. Pasán , A. H. Daranas , R. Carrillo , Angew. Chem., Int. Ed. 2021, 60, 18783; 10.1002/anie.202106230 PMC8457238 34085747

[21] D. Andrade‐Acuña , J. G. Santos , W. Tiznado , Á. Cañete , M. E. Aliaga , J. Phys. Org. Chem. 2014, 27, 670.

[22] D. Tanini , A. Capperucci , Adv. Synth. Catal. 2021, 363, 5360.

[23] M. J. Tucker , J. R. Courter , J. Chen , O. Atasoylu , A. B. Smith Iii , R. M. Hochstrasser , Angew. Chem., Int. Ed. 2010, 49, 3612. 10.1002/anie.201000500 PMC2903731 20391451

[24] S. G. Tolshchina , R. I. Ishmetova , N. K. Ignatenko , A. V. Korotina , I. N. Ganebnykh , V. A. Ol'shevskaya , V. N. Kalinin , G. L. Rusinov , Russ. Chem. Bull. 2011, 60, 985.

[25] a) K. Banerjee , D. Bhattacherjee , S. K. Mahato , A. Sufian , K. P. Bhabak , Inorg. Chem. 2021, 60, 12984; 34369772 10.1021/acs.inorgchem.1c01410

[26] O. Chaudhuri , J. Cooper‐White , P. A. Janmey , D. J. Mooney , V. B. Shenoy , Nature 2020, 584, 535. 32848221 10.1038/s41586-020-2612-2 PMC7676152

[27] M. J. Tucker , M. Abdo , J. R. Courter , J. Chen , A. B. Smith , R. M. Hochstrasser , J. Photochem. Photobiol. A: Chem. 2012, 234, 156. 22577259 10.1016/j.jphotochem.2012.02.014 PMC3345895

[28] a) W. Li , Y. Bei , X. Pan , J. Zhu , Z. Zhang , T. Zhang , J. Liu , D. Wu , M. Li , Y. Wu , J. Gao , Biomater. Res. 2023, 27, 49; 37202774 10.1186/s40824-023-00367-w PMC10193707

[29] K. Zheng , Y. Tong , S. Zhang , R. He , L. Xiao , Z. Iqbal , Y. Zhang , J. Gao , L. Zhang , L. Jiang , Y. Li , Adv. Funct. Mater. 2021, 31, 2102599.

[30] a) B. Jia , G. Li , E. Cao , J. Luo , X. Zhao , H. Huang , Mater. Today Bio 2023, 19, 100582; 10.1016/j.mtbio.2023.100582 PMC9988584 36896416

[31] a) N. M. Brown , L. O. White , E. L. Blundell , S. R. Chown , R. R. Slade , A. P. MacGowan , D. S. Reeves , J. Antimicrob. Chemother. 1993, 32, 117; 10.1093/jac/32.1.117 8226402

