# Supplementary material for: Advanced Multifunctional Hydrogels for Enhanced Wound Healing through Ultra‐Fast Selenol‐SNAr Chemistry
Source: Adv Sci (Weinh). 2024 Apr 22;11(21):2400898. doi: 10.1002/advs.202400898 (PMC11151048; doi:10.1002/advs.202400898)
Supplement: Supplementary file 1 — Supporting Information [file ADVS-11-2400898-s002.pdf]

ADVANCED SCIENCE
Open Access

Supporting Information

for *Adv. Sci.*, DOI 10.1002/advs.202400898

Advanced Multifunctional Hydrogels for Enhanced Wound Healing through Ultra-Fast Selenol-S_NAr Chemistry

Yan Wu, Ying Bei, Wenjing Li, Weihong Lu, Jian Zhu, Zhengbiao Zhang, Tinglin Zhang, Sen Liu, Kaiyuan Chen, Hong Jin, Luxin Li, Meng Li*, Jie Gao* and Xiangqiang Pan**

Supporting Information
©Wiley-VCH 2021
69451 Weinheim, Germany

Advanced Multifunctional Hydrogels for Enhanced Wound Healing through Ultra-Fast Selenol-S_NAr Chemistry

Yan Wu[b,*,1], Ying Bei[b,f,1], Wenjing Li[a,1], Weihong Lu[a], Jian Zhu[a], Zhengbiao Zhang[a], Tinglin Zhang[c], Sen Liu[b], Kaiyuan Chen[b], Hong Jin[b], Luxin Li[b], Meng Li[d,*], Jie Gao[c,e,*],Xiangqiang Pan[a, b,*]

DOI: 10.1002/anie.2021XXXXX

[a] State and Local Joint Engineering Laboratory for Novel Functional Polymeric Materials, Jiangsu Key Laboratory of Advanced Functional Polymer Design and Application, College of Chemistry, Chemical Engineering and Materials Science, Soochow University
Suzhou 215123, China
E-mail:panxq@suda.edu.cn

[b] College of Life Science, Mudanjiang Medical University
Mudanjiang 157011, China

[c] Changhai Clinical Research Unit, Shanghai Changhai Hospital, Naval Medical University
Shanghai 200433, China

[d] Department of dermatology Shanghai Children's Medical Center, School of Medicine, Shanghai Jiao Tong University
Shanghai 200010, China

[e] Shanghai Key Laboratory of Nautical Medicine and Translation of Drugs and Medical Devices, Shanghai 200433, China

[f] Hainan Aademy of Medical Sciences, Hainan Medical University, Hainan 571199, China

[1] These authors contributed equally to this paper.

* Corresponding authors. Xiangqiang Pan. State and Local Joint Engineering Laboratory for Novel Functional Polymeric Materials, Jiangsu Key Laboratory of Advanced Functional Polymer Design and Application, College of Chemistry, Chemical Engineering and Materials Science, Soochow University, Suzhou 215123, China. E-mail: panxq@suda.edu.cn.

* Corresponding authors. Jie Gao. Changhai Clinical Research Unit, Shanghai Changhai Hospital, Naval Medical University, Shanghai 200433, China. E-mail: gaojiehighclea@smmu.edu.cn.

* Corresponding authors. Meng Li. Department of Dermatology, Shanghai Ninth People's Hospital, Shanghai Jiaotong University. Shanghai 200010, China. lemonlives_dr@163.com.

* Corresponding authors.Yan Wu. College of Life Science, Mudanjiang Medical University, Mudanjiang 157011, China. E-mail: wuyan@mdjmu.edu.cn

Table of Contents

Table of Contents 2
Experimental Procedures 3
- Instruments 3
- Materials 3
- Model reaction of selenol and dichloromethyltetrazine 3
- Synthesis of SeH and PEG-Se_2 hydrogels 3
- Synthesis of Se-Tz hydrogels 4
- Rheological measurements 4

Hydrogels' antibacterial characteristics *in vitro* 4
Biocompatibility assay 4
Cell proliferation and migration assays 4
Animal studies 5
In vivo antibacterial evaluation 5
In vivo diabetic wound healing evaluation 5
Results 6
Figure S1 The rapid reaction process of selenol and DT.. 6
Figure S2 Model reaction of alkyl selenol with dichlorometetrazine. 7
Figure S3 Properties of hydrogels obtained by reaction of selenol with dichlorometetrazine in different proportions. 8
Figure S4 Degradation process of Se-Tz small molecules. 9
Figure S5 Rheological characterization of the hydrogels.. 10
Figure S6 Thixotropy of hydrogels.. 10
Figure S7 Fatigue testing of hydrogels.. 11
Figure S8 Self-healing performance test of hydrogels.. 12
Figure S9 Photodegradation properties of hydrogels. 12
Figure S10 Antibacterial properties of hydrogels. 13
Figure S11 Proliferation of hydrogels and Met. 14
Figure S12 Migration efficacy of hydrogels and Met.. 15
Figure S13 Histological analysis of diabetic wounds treated with hydrogels and Met... 16
Figure S14 Histological analysis of diabetic wounds treated with hydrogels and Met... 17
References 17
Author Contributions 17

Experimental Procedures

Instruments

Nuclear magnetic resonance (NMR)

^{1}H-NMR and ^{13}C-NMR spectra were recorded in DMSO-d_6 or $CDCl_3$ on a Bruker Avance 300 at 300/75 MHz or on a Bruker Avance 400 at 400/100 MHz. Chemical shifts are presented in parts per million (δ) relative to $CDCl_3$ (7.26 ppm in ^{1}H- and 77.00 ppm in ^{13}C-NMR, respectively) as an internal standard. Coupling constants (J) in ^{1}H-NMR are given in Hz. The resonance multiplicities are described as *s* (single), *d* (doublet), *t* (triplet), q (quartets) or *m* (multiplet).

Fourier-transform infrared spectroscopy (FT-IR)

Fourier transform infrared spectra were measured on a Bruker TENSOR 27 FT-IR spectrometer (KBr disk).

Liquid chromatography–mass spectrometry (LC-MS)

LC-MS spectra were obtained on a Bruker micro TOF-QIII, and the samples were dissolved in HPLC methanol.

X-ray photoelectron spectroscopy (XPS)

X-ray photoelectron spectroscopy was carried out using an ESCALAB250XiII.

Rheological measurements

Rheological measurements were made by the HAAKE Rheometer (RS 6000) instrument, with the rotor and the sample table spaced at 1 mm and the rotor being a parallel plate with a diameter of 20 mm.

Ultraviolet-visible spectroscopy (UV-vis)

Ultraviolet-visible spectra were determined on a Shimadzu UV-2600 spectrophotometer at 25°C.

High performance liquid chromatography (HPLC)

High performance liquid chromatography (HPLC) was performed on a Thermofisher Ultimate3000 high performance liquid chromatography at 35°C. Acetonitrile and water were used as mobile phase, the ratio was 4:6, and the retention time was 30 min. The detection wavelength is 254 nm.

Materials

4-arm-PEG-NH_2 (M_n = 10 kDa) and arm-PEG-SH (M_n = 10 kDa) were procured from Shanghai Peng Sheng Biotechnology (Shanghai, China). Using a one-pot and two-step technique, γ-selenobutylacetone (γ-SBL) was synthesized from 4-chlorobutyryl chloride and newly prepared NaSeH.[1] Dichlorotetrazine (97%) was procured from Shanghai Haohong Biotechnology (Shanghai, China). Metformin Hydrochloride (98%) was purchased from Macklin (Shanghai, China). *N*-propylamine (99%), Tributylphosphine was procured from Aladdin (Shanghai, China).

Model reaction of selenol and dichloromethyltetrazine

N-propylamine (118 mg, 2 mmol) was dissolved in 10 mL THF, oxygen was removed by argon for 10 min, and then γ-selenbutylactone (315 mg, 2.1 mmol) was injected into the syringe, stirring at room temperature for 8 h. Dichlorometetrazine (166 mg, 1.1 mmol) was added to the above solution, stirring reaction was carried out, and TLC was followed up. After spinning the solvent and dissolving it with deuterated chloroform ($CDCl_3$) as solvent, nuclear magnetic hydrogen spectrometry and high performance liquid chromatography were performed.

Synthesis of SeH and PEG-Se_2 hydrogels

The preparation of 4-arm polyethylene glycol with selenol (4-arm-PEG-SeH) followed the protocol outlined in our earlier research. One gram of 4-arm-PEG-NH_2 was added to 4 mL of deionized water. Thereafter, SBL was added to the solution at a molar ratio of 4:1 (SBL to 4-arm-PEG-NH_2), followed by continuous stirring at 25°C for 8 h, resulting in the successful synthesis of 4-arm-PEG-SeH. The solution was placed in the air for oxidative crosslinking for 24 h to prepare PEG-Se_2 hydrogels.

Synthesis of Se-Tz hydrogels

The pH was adjusted to 8.5 by adding triethylamine to the obtained selenium-containing alcohol aqueous solution (10 mL, in which selenium-alcohol-modified four-arm polyethylene glycol had a mass fraction of 20 wt%). According to the molar ratio of selenol and dichlorometetrazine as 4:1,2:1,1:1 and 1:2, respectively, dichlorometetrazine was weighed, dissolved in 0.2 mL DMSO, added to the above solution, fully stirred until the gel was formed, and hydrogels P1, P2, P3 and P4 were obtained after exposure to air oxidation for 48 h.

Rheological measurements

All rheological measurements were performed at 37 °C to simulate the human environment. The distance between the rotor and the sample stage was 1 mm when using a 25-mm parallel plate for measurement and testing. The strain sweep settings were as follows: frequency, 1 Hz; strain sweep,1–3000%. The frequency sweep settings were as follows: strain, 1%; frequency sweep, 1–100 Hz. The time sweep test was performed at a frequency of 1 Hz, strain of 1% and sweep time of 300 s. In addition, the self-healing performance of the hydrogels was tested *via* rheological analysis. The hydrogels were scanned for 120 s alternately under strain conditions of 1% and 3000%, with a total of 3 cycles. After a cycle was completed, another cycle of 120 s was run. For the low-shear measurement at 0.1% strain, the frequency remained constant at 10 Hz during the entire process.

Hydrogels' antibacterial characteristics *in vitro*

Hydrogels and Tz (Tz, Se-Tz and Se-Tz@Met groups containing 0.25 mmol mL^{-1} of cyano group) were introduced into 1 mL of bacterial solution (*S. aureus, E. coli* and *MRSA*, 1 × 10^7 CFU mL^{-1}), and the mixture was either subjected to green light (520 nm, 1 W cm^{-2}) or placed in darkness. Subsequently, the solution was diluted 1000 times, and 50 μL of the solution was extracted before being placed onto a culture plate. After 16 h of culture, images were captured and analyzed.

Biocompatibility assay

The biocompatibility of hydrogels and Tz was evaluated *via*calcein acetoxymethyl ester (calcein-AM) and propidium iodide (PI) double-staining and methylthiazoletetrazolium (MTT) assays. First, double labeling with calcein-AM and PI, as reported earlier, was performed to determine cell viability.[2] NIH-3T3 and HUVEC cells were treated with hydrogels and Tz before incubation at 37 °C for 24 h (all Se-Tz and Tz contained 0.25 mmol·mL^{-1}cyano group). Samples in the groups treated with green light (GL) were irradiated with GL (520 nm, 1 W cm^{-2}, 15 min, VCLHLGD0025017, Blueprint, Beijing, China). NIH-3T3 and HUVEC cells were stained for 40 min with red (PI) and green (calcein-AM) fluorescent dyes at 37 °C and imaged under a live cell imaging system (DMI6000B; Feica, Wetzlar, Germany). Second, cell viability was evaluated *via* the MTT assay.Briefly, NIH-3T3 and HUVEC cells were seeded in a 96-well plate (5 × 10^3 cells/well). Following cell attachment, the hydrogels (0–781 μg·mL^{-1}) and Tz (0–60 mg·mL^{-1}) were added to the cells. The GL-treated groups were illuminated with GL (520 nm, 1 W cm^{-2}, 15 min, VCLHLGD0025017, Blueprint, Beijing, China). After 24 h of incubation, the cells were washed and incubated for 4 h at 37°C in fresh medium containing MTT reagent. Finally, the absorbance of formazan crystals dissolved in DMSO was measured at 490 nm using a microplate reader (SpectraMax M3, Molecular Devices, California, USA).

Cell proliferation and migration assays

NIH-3T3 purchased from ATCC were cultured in DMEM medium supplement with 10% FBS and 1% penicillin−streptomycin and kept in a 37 °C humidified incubator contained 5% CO_2. The NIH-3T3 and HUVEC proliferation stimulated by Se-Tz@Met scaffold dressing was evaluated by MTT. For cell migration assay and tube formation assay, NIH-3T3 and HUVECs were firstly co-cultured with FEP@exo hydrogel in the upper chamber of the transwell insert and culture medium with 1% FBS was added into the lower chamber. After 24 h of culture, the cells on the membrane of the upper chamber were carefully removed with a cotton swab. Then, cells migrated into the lower chamber was fixed with 4% paraformaldehyde and stained with crystal violet. An inverted microscope (Nikon, FHEIPSE Ti, Japan) was used to observe the stained cells.

Animal studies

Male Institute of Cancer Research (ICR) mice (weight, 25–30 g; age, 6–8 weeks) were provided by Beijing Vital River Laboratory Animal Technology Co., Ltd. (Beijing, China). All animal experiments were approved by and conducted following the guidelines of the Ethics Committee on Animals of Mudanjiang Medical University (Mudanjiang, China) (approval number: 20230426-57). Animal experiments were performed in a specific pathogen-free laboratory. Mice were housed in an environment with a relative humidity of 50–60%, a temperature of 20–24°C, and a 12-h dark/light cycle. Finally, they were euthanized with carbon dioxide.

***In vivo* antibacterial evaluation**

After two days of acclimation, eight groups (three mice each) were established at random: control, light, Tz, Tz + GL, Se-Tz, Se-Tz + GL, Se-Tz@Met and Se-Tz@Met + GL groups. Each mouse had a wound made on its back with a diameter of 6 mm. After the injection of 0.1 mL of bacterial solution (10^7 CFU·mL^{-1}), mice in various groups received different care for their wounds. Next, the wounds were covered with 0.1 mL PBS in the control and light groups. In the Tz and Tz + GL groups, the wounds were covered with 0.1 mL of Tz (0.25 mmol·mL^{-1} of cyano group). In the Se-Tz and Se-Tz + GL groups, the wounds were covered with 0.1 mL of Se-Tz (containing 0.25 mmol·mL^{-1} of cyano group). In the DSeP@PB and DSeP@PB + RL groups, the wounds were covered with 0.1 mL DSeP@PB (containing 0.25 mmol·mL^{-1} of cyano group). Every day, the GL groups were subjected to green light (520 nm, 1 W·cm^{-2}, 15 min). On day 7, the wound was cut to observe the infection. Then， tissue fluid smeared by a sterile cotton stick from the wound site was mixed with 1 mL of bacterial medium. Thereafter, 50 μL of the solution was extracted and spread on a plate. After 16 h of culture, images were captured and analyzed.

***In vivo* diabetic wound healing evaluation**

To establish type 1 diabetes in male ICR mice, the mice were fasted overnight before having their abdominal cavity injected with 100 mg kg^{-1} of streptozotocin (STZ). This was followed by an injection of 50 mg kg^{-1} once daily for two days. One week later, blood glucose levels were monitored, and diabetes status was determined based on whether blood glucose levels were higher than 16.7 mM. Each mouse had two circular full-thickness cutaneous lesions (diameter = 6 mm) made on its back. Seven groups of mice were established at random and treated with 200 μL of Se-Tz hydrogels, Met, Se-Tz@Met or PBS (control). Mice in the Se-Tz + GL, Met + GL and Se-Tz @Met + GL groups were illuminated with 520-nm GL for 15 min with a power density of 1 W cm^{-2} (the Se-Tz@Met and Met groups contained 100 μg·mL^{-1} of Met). On days 0, 3, 6, and 12, digital images of the wounds were captured using a digital camera to track their progress as they healed. The following formula was used to determine the wound healing rate: wound healing rate = (wound area at day 0 − wound area at a certain day)/wound area at day 0. A histological investigation was performed using H&E and Masson's trichrome stains, as per the manufacturer’s instructions. A Nikon FHEPSE 80i microscope (Nikon, Japan) was employed to examine the slides. The immunostaining of CD86, CD206 and Ki67 was performed to evaluate the Inflammatory response and proliferation during the healing period, respectively.

Results

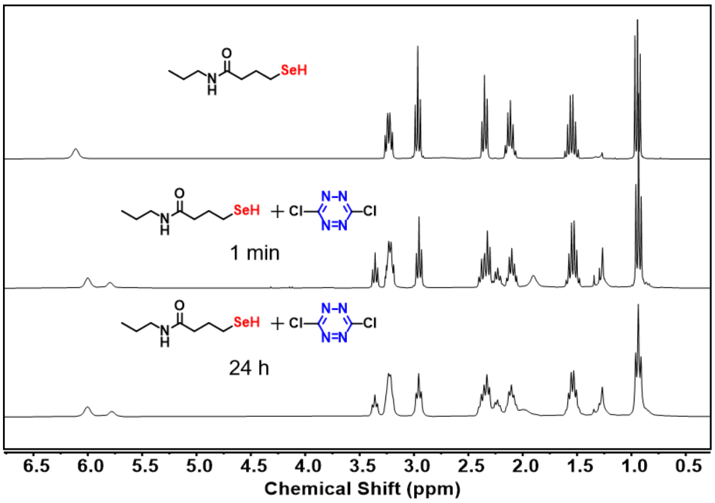

Figure S1. The rapid reaction process of selenol and DT. 1H NMR spectra of selenol, selenol reacting with dichloromethane for 1 min and 24 h.

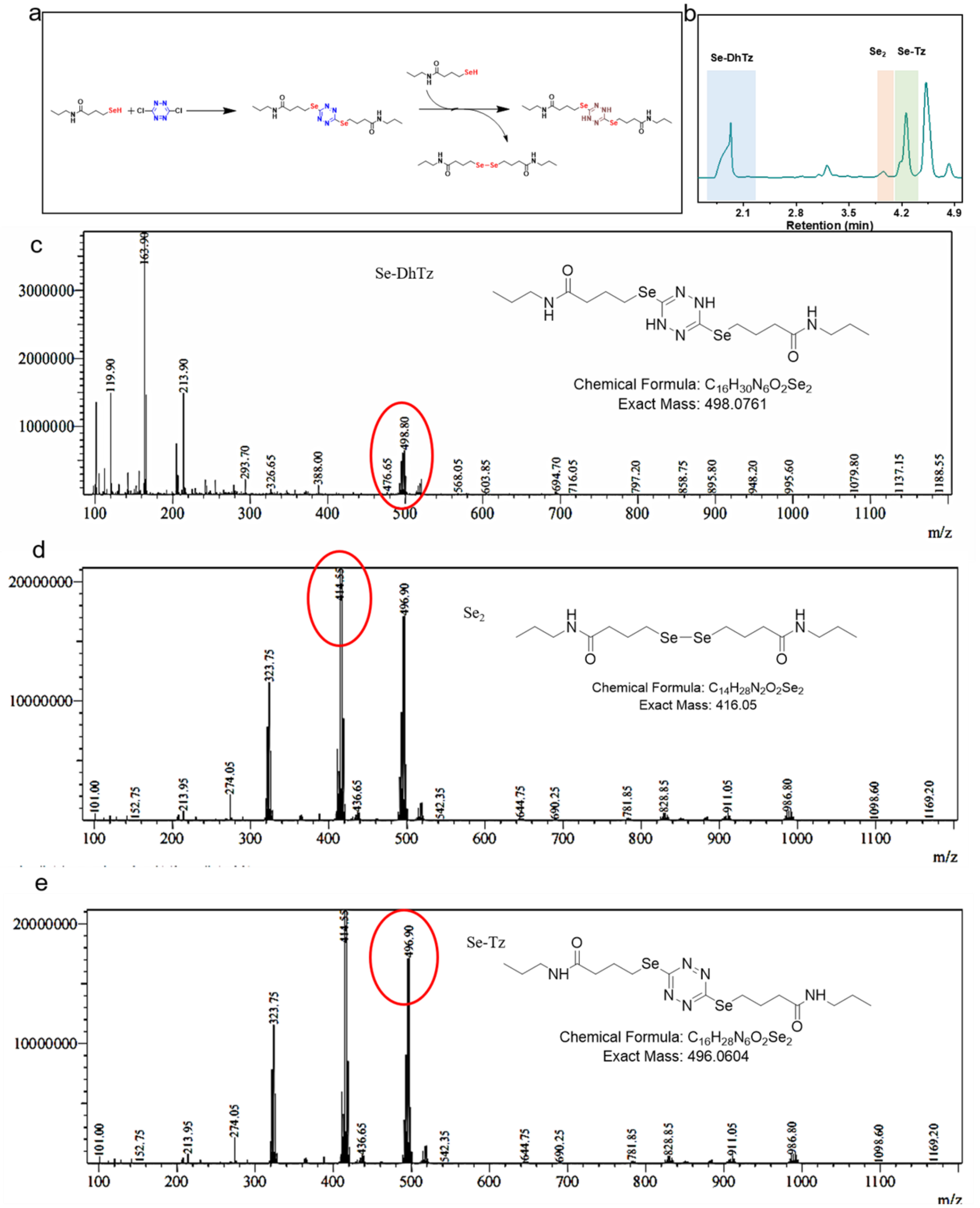

Figure S2. Model reaction of alkyl selenol with DT. (a)the reaction of selenol and DT. (b) HPLC curve of the reaction of selenol and DT (1.2:1). (c), (d), (e) LC-MS spectra of Se-DhTz, Se_2 and Se-Tz.

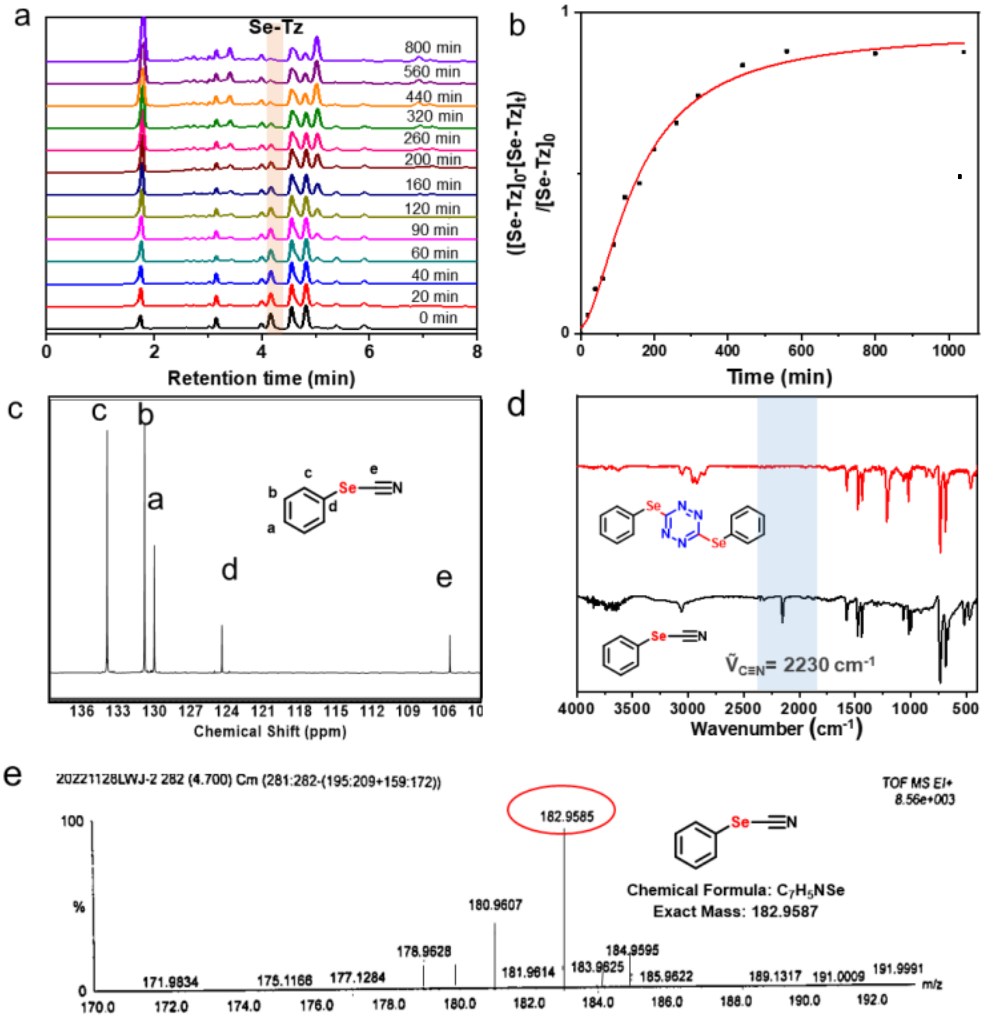

Figure S3. Degradation process of Se-Tz small molecule. (a) he photodegradation process of Se-Tz small molecule tracked by HPLC. (b) Degradation kinetics of Se-Tz. (c) ^{13}C NMR spectrum of photodegradation products. (d) FT-IR spectra of photodegradation products and Se-Tz (e) GCT-TOF-MS.of photodegradation products.

a

Entry	$[4arm\text{-}PEG\text{-}SeH]_0 : [DT]_0$	State
P1	4 : 1	Hydrogel
P2	2 : 1	Hydrogel
P3	1 : 1	Hydrogel
P4	1 : 2	Liquid

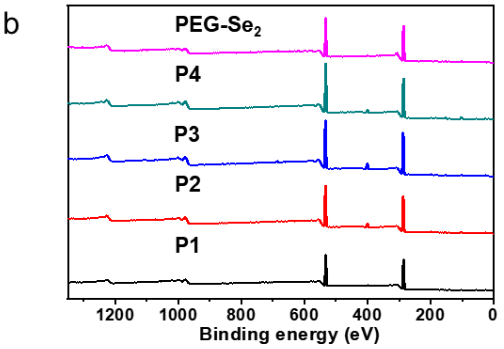

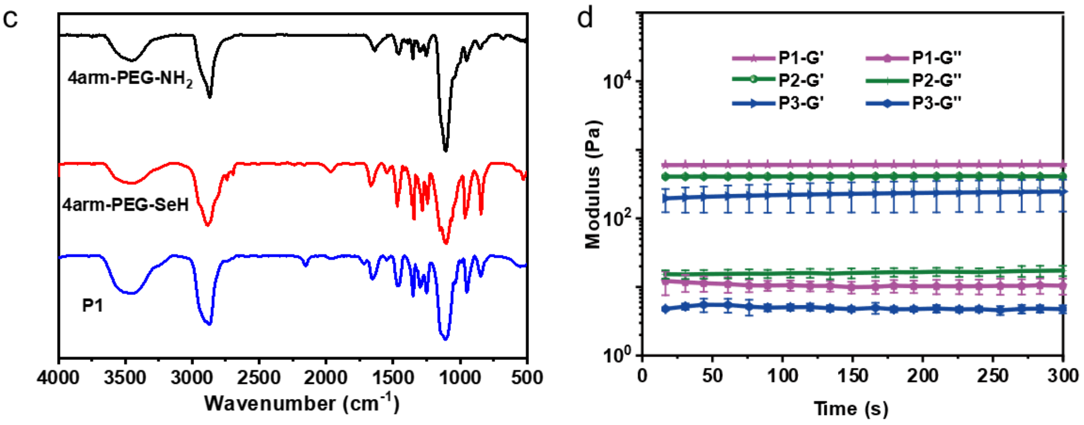

Figure S4. Properties of hydrogels obtained by reaction of selenol with DT in different proportions. (a) Nomenclature and state of reactions of different proportions of selenol with dichlorometetrazine. (b) XPS spectra of $PEGSe_2$, P1, P2, P3 and P4. (c) The infrared spectrum of $4arm\text{-}PEG\text{-}NH_2$, 4arm-PEG-SeH and P1. (d) Rheological behaviors of hydrogels.Due to an excessive presence of monosubstituted tetrazine in the polymer structure at a 2:1 ratio, the gelation ability of the reaction system diminishes significantly, thus limiting further discussion on this matter within the scope of this paper

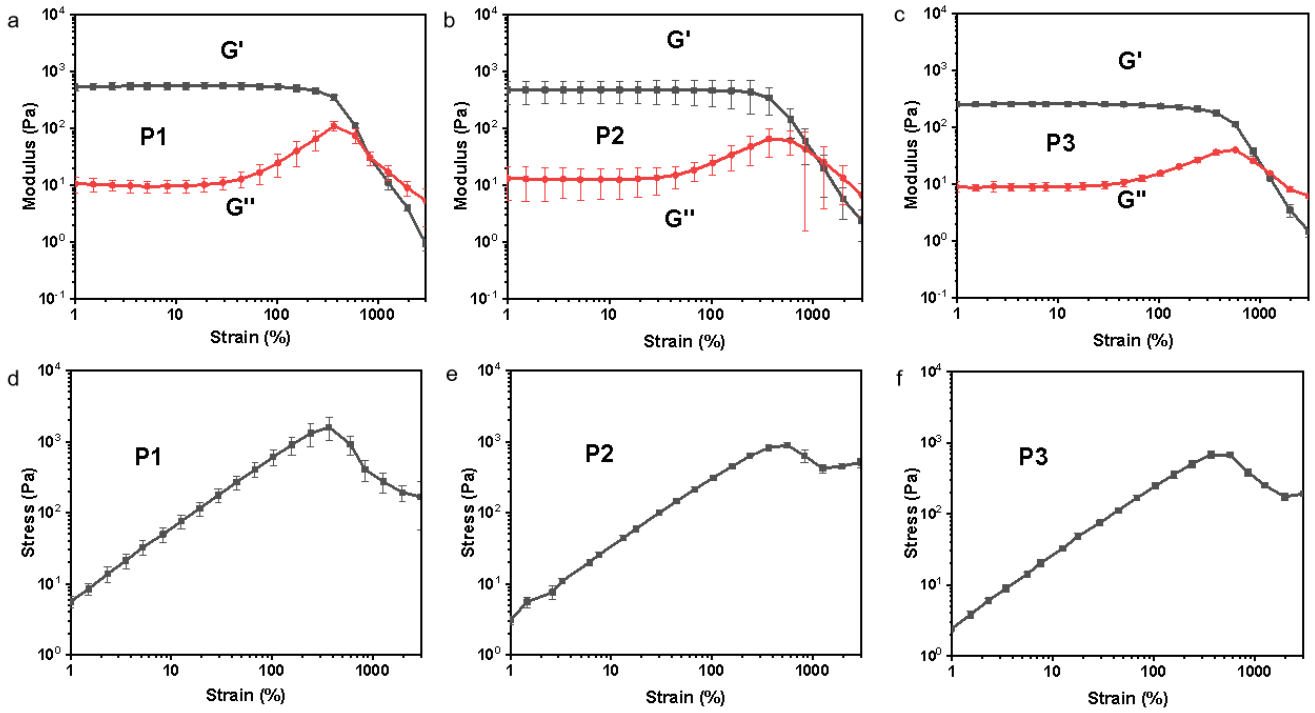

Figure S5. Rheological characterization of the hydrogels. (a), (b), (c) Strain sweep of P1, P2, P3. (d), (e), (f) Stress–strain profiles of P1, P2, P3. Data are presented as means ± SD (n = 3).

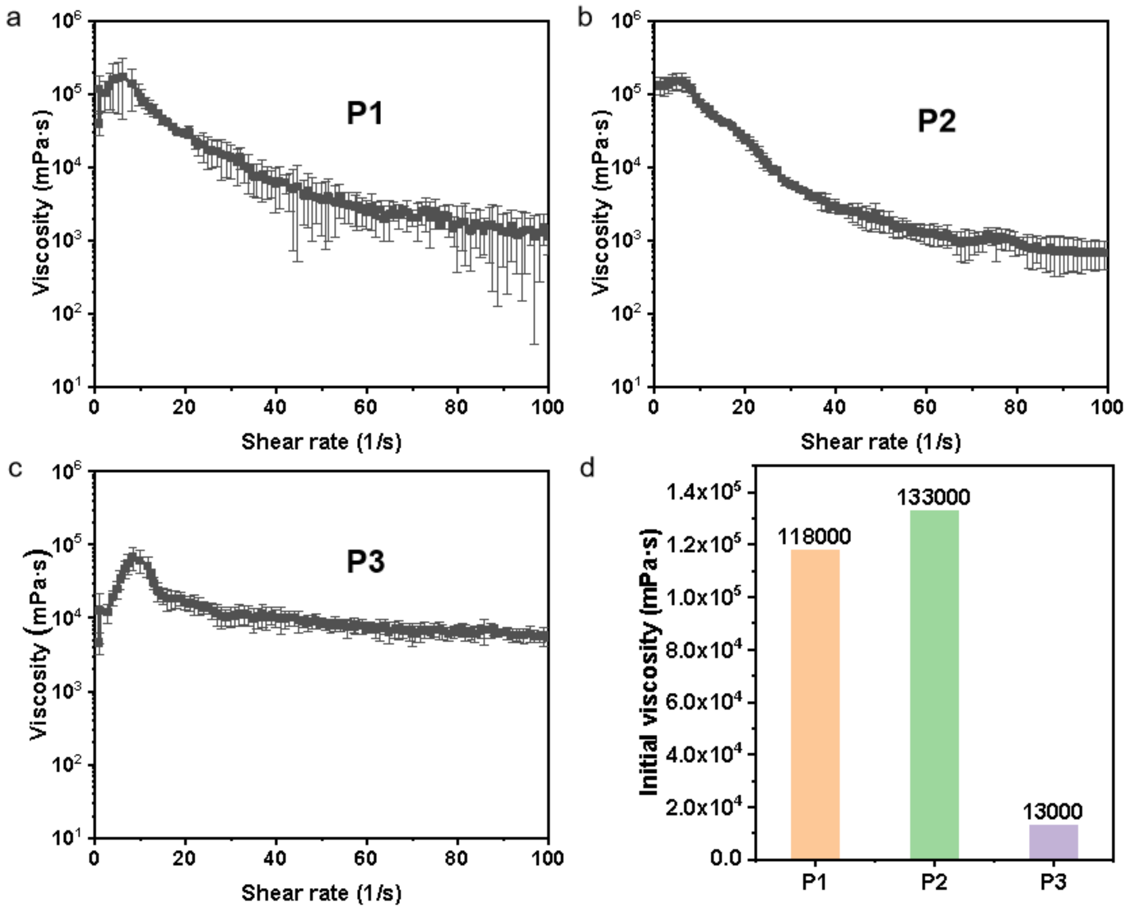

Figure S6 Thixotropy of hydrogels. (a), (b), (c) Relationship between viscosity of P1, P2, P3 and shear rate. (d) The initial viscosity of hydrogels. Data are presented as means ± SD (n = 3).

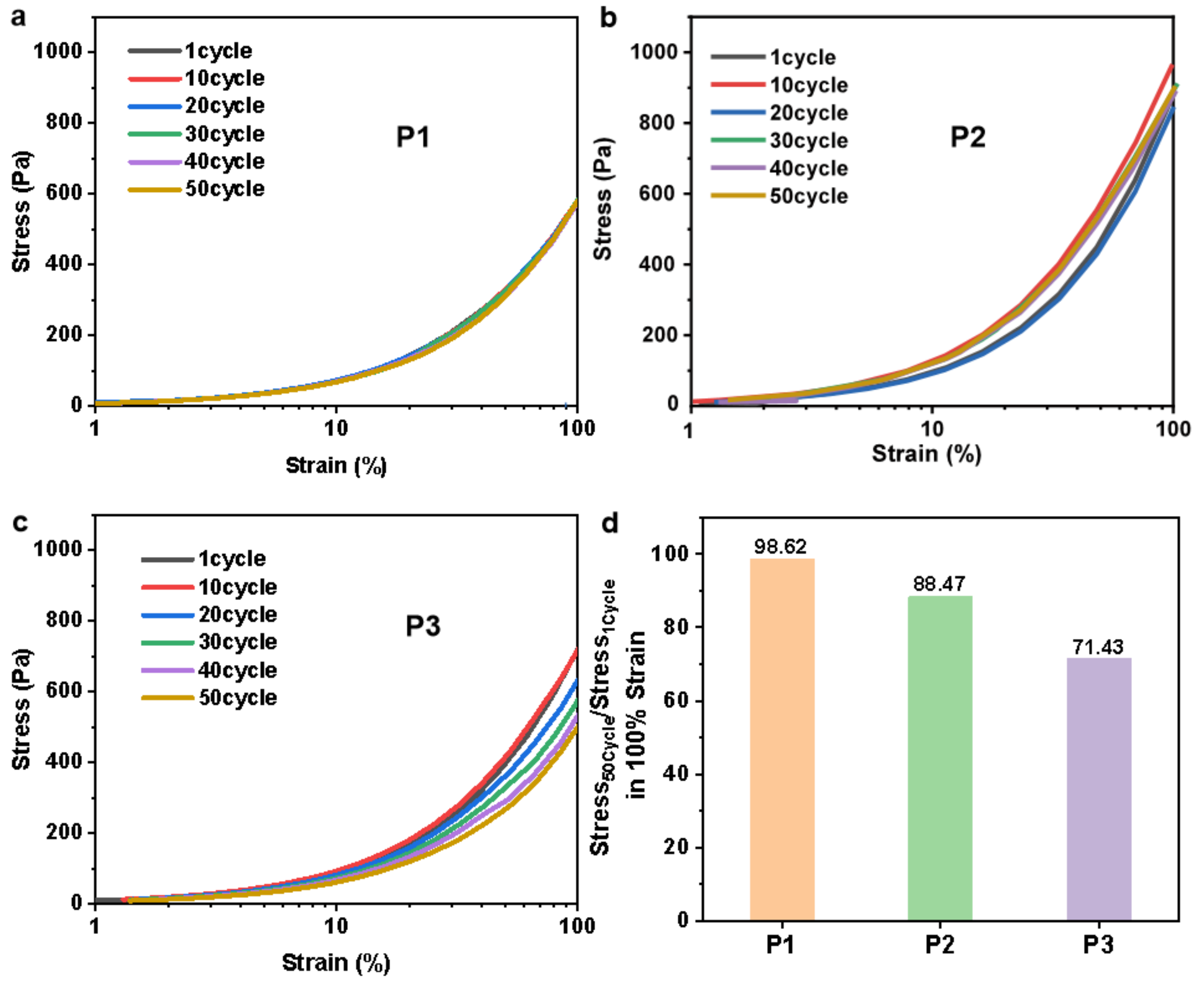

Figure S7. Fatigue testing of hydrogels. (a), (b), (c)The cycling curves of stress–strain for P1, P2, P3. (d) The ratio of stress at 50 cycle to stress at 1 cycle in 100% stress.

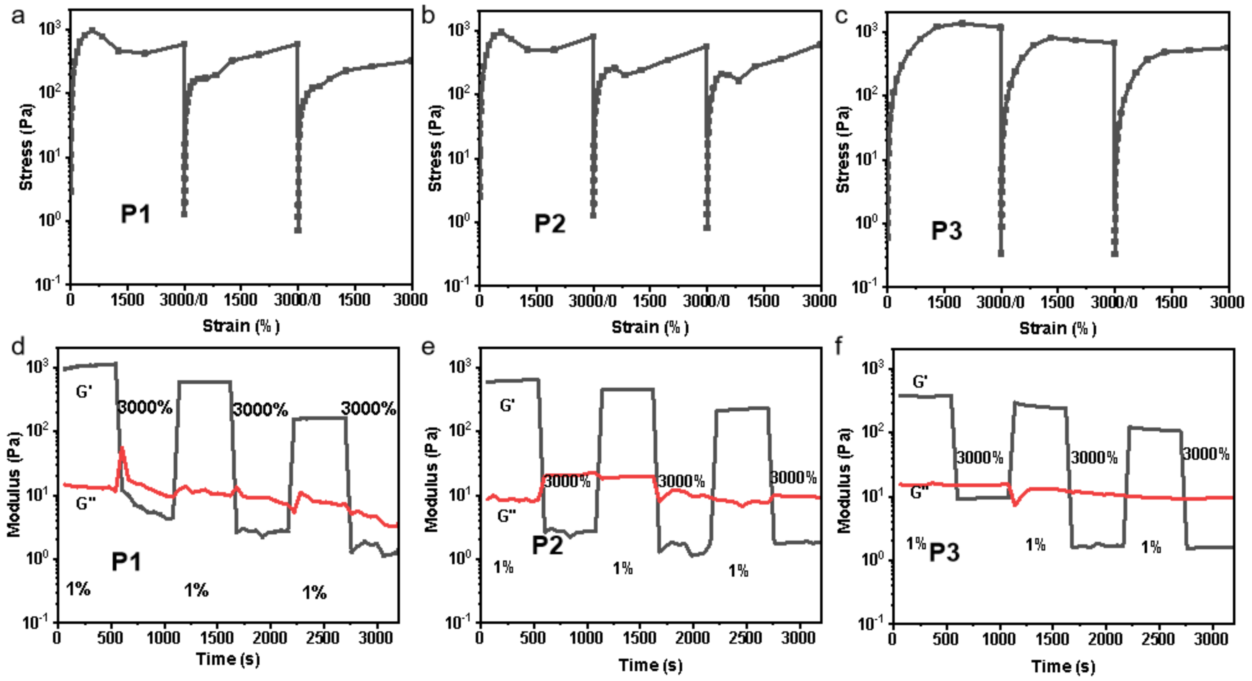

Figure S8. Self-healing performance test of hydrogel P2. (a), (b), (c) The stress–strain profiles of hydrogels assessed by the strain sweep test from 1% to 1000% for 3 cycles. (d), (e), (f) The elastic modulus (G') and viscous modulus (G'') of hydrogels assessed by the strain sweep test from 1% to 1000% for 3 cycles

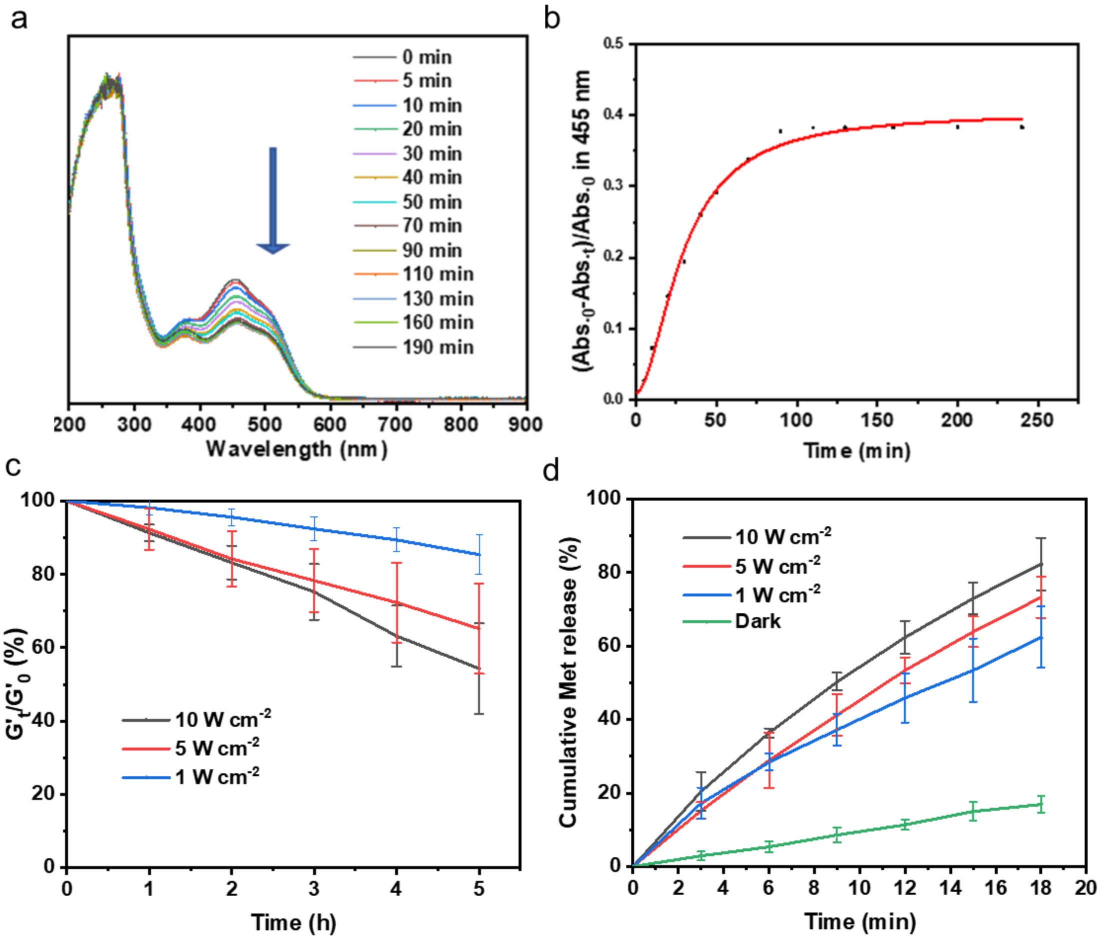

Figure S9. Photodegradation properties of hydrogels. (a) Ultraviolet-visible absorption curve of P1 hydrogel under 520 nm green light irradiation (10 W cm^{-2}); (b) Degradation kinetics of P1 hydrogel under 520 nm green light irradiation (10 W cm^{-2}). (c) The relation between the ratio of the energy storage modulus (G'_t) of different hydrogels to the initial energy storage modulus (G'_0) with time under 520 nm green light (three different powers). (d) The drug release profile of Se-Tz hydrogel loaded with metformin was exposed to 520 nm for a duration of 10 min. Data are presented as means ± SD (n = 3).

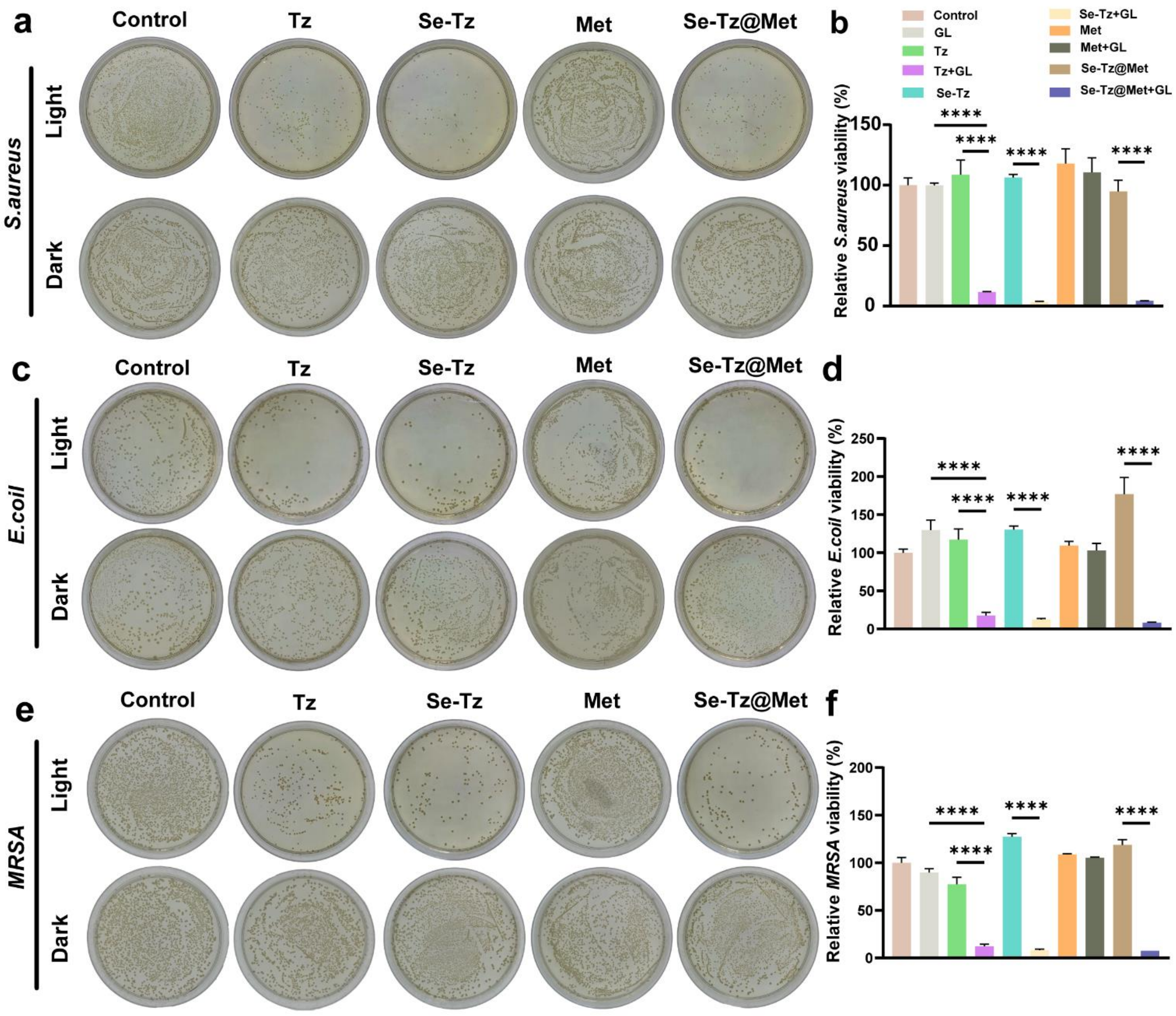

Figure S10. Antibacterial properties of hydrogels. (a) (c) and (e) Photographs of plates showing the bacterial colony formation assay in the presence or absence of green light irradiation. (b) (d) and (f) Relative viability of bacterial colonies calculated from (a) (c) and (e), respectively. *$P < 0.05$; **$P < 0.01$; ***$P < 0.001$; ****$P < 0.0001$. Data are presented as the means ± SDs (n = 3)

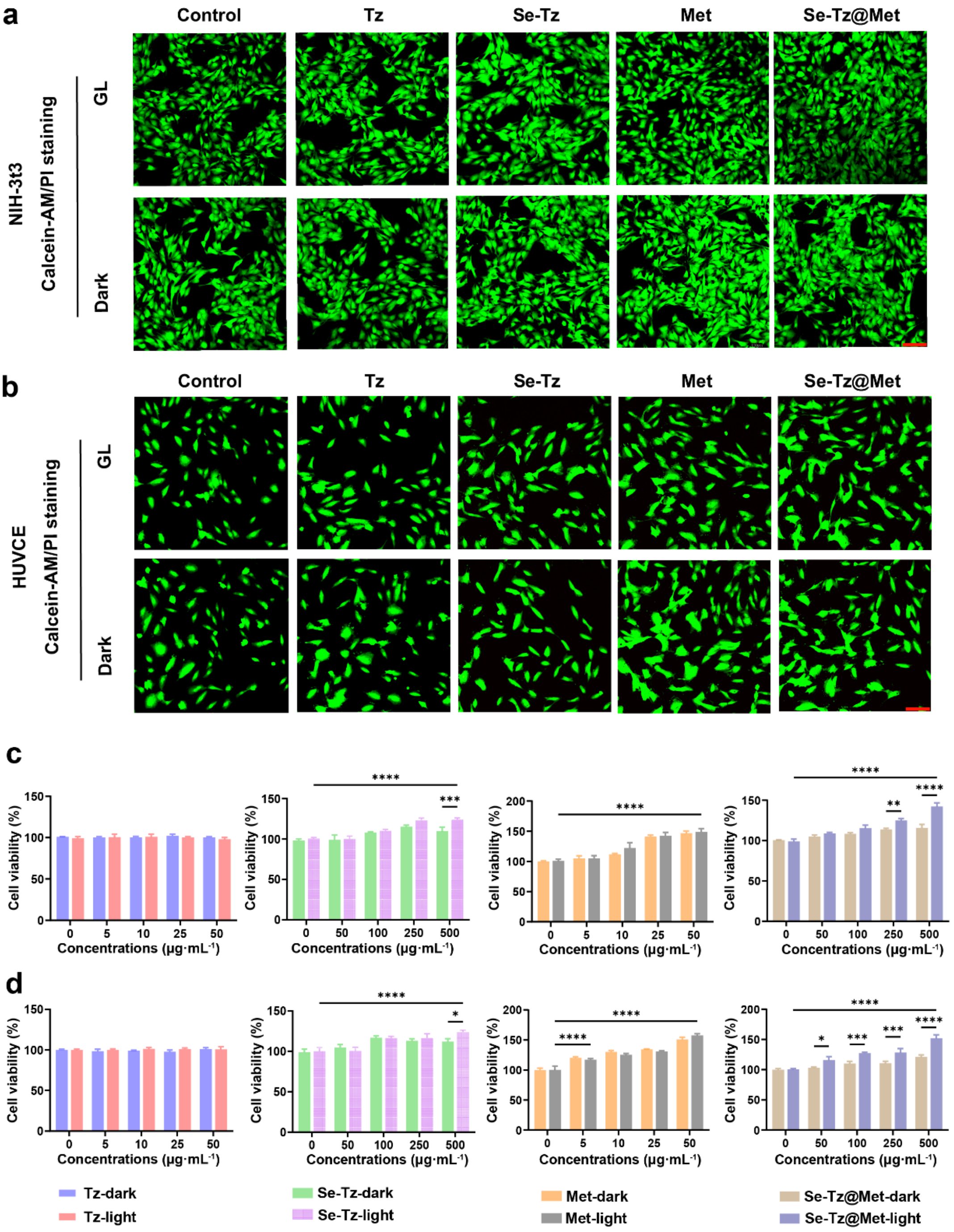

Figure S11. Proliferation efficacy of hydrogels and Met. (a, b) NIH-3T3 and HUVEC cells were stained with calcein-AM (green) and propidium iodide (PI, red) after different treatments for 24 h. Bars represent 100 μm. (c) Cell viability of NIH-3T3 evaluated by the MTT assay after different treatments for 24 h. (d) Cell viability of HUVEC evaluated by the MTT assay after different treatments for 24 h. *P< 0.05; **P < 0.01; ***P < 0.001; ****P< 0.0001. Data are presented as the means ± SDs (n = 3).

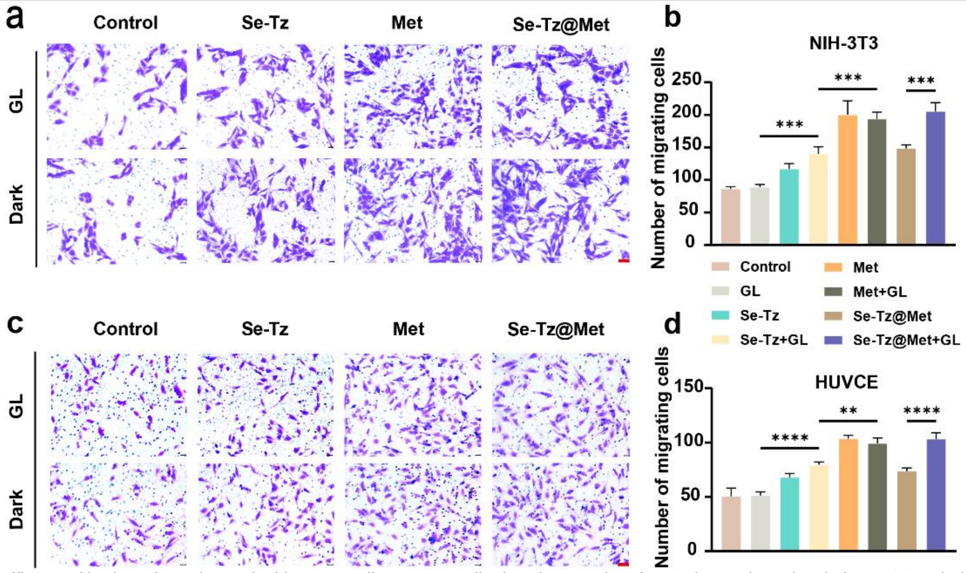

Figure S12. Migration efficacy of hydrogels and Met. (a, b) Transwell NIH-3T3 cell migration results after 24 h coculture (scale bar: 100 μm); (c, d) Transwell HUVEC cell migration results after 24 h coculture (scale bar: 50 μm). *P< 0.05; **P< 0.01; ***P< 0.001; ****P< 0.0001. Data are presented as the means ± SDs (n = 3).

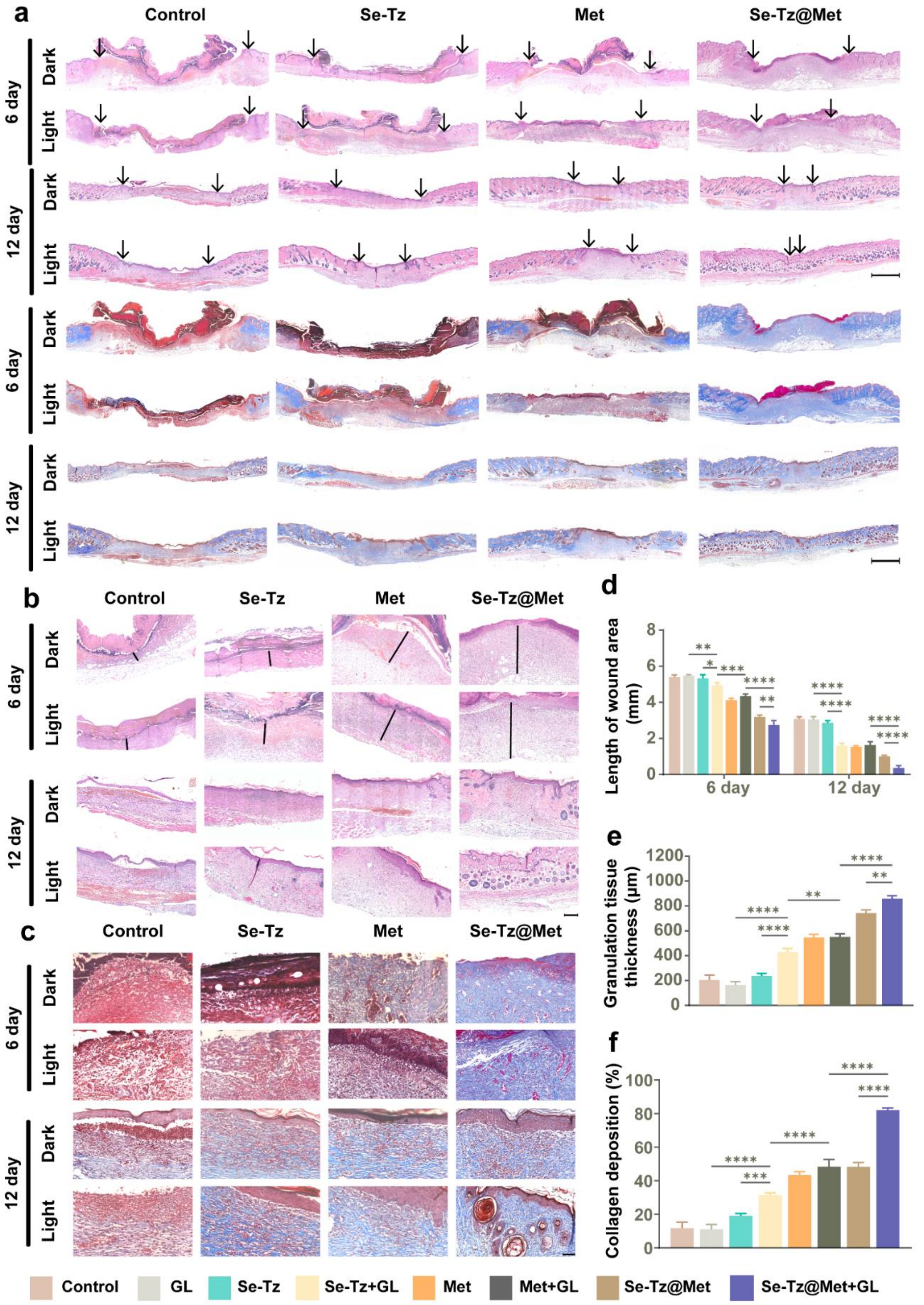

Figure S13. Histological analysis of diabetic wounds treated with hydrogels and Met. (a) H&E and Masson staining images of diabetic wounds of mice with each treatment on days 6 and 12. Bars represent 1 mm. (b) H&E staining images of diabetic wounds of mice with each treatment at days 6 and 12. Bars represent 100 μm. (c)Masson staining images of diabetic wounds of mice with each treatment on days 6 and 12. Bars represent 50 μm. (d) Quantification of the length of the wound area at day 6 and 12. (e) Quantification of granulating tissues at day 6. (f) Quantitative analysis of collagen deposition at day 12. **P*< 0.05; ***P*< 0.01; ****P*< 0.001; *****P*< 0.0001. Data are presented as the means ± SDs (n = 3)

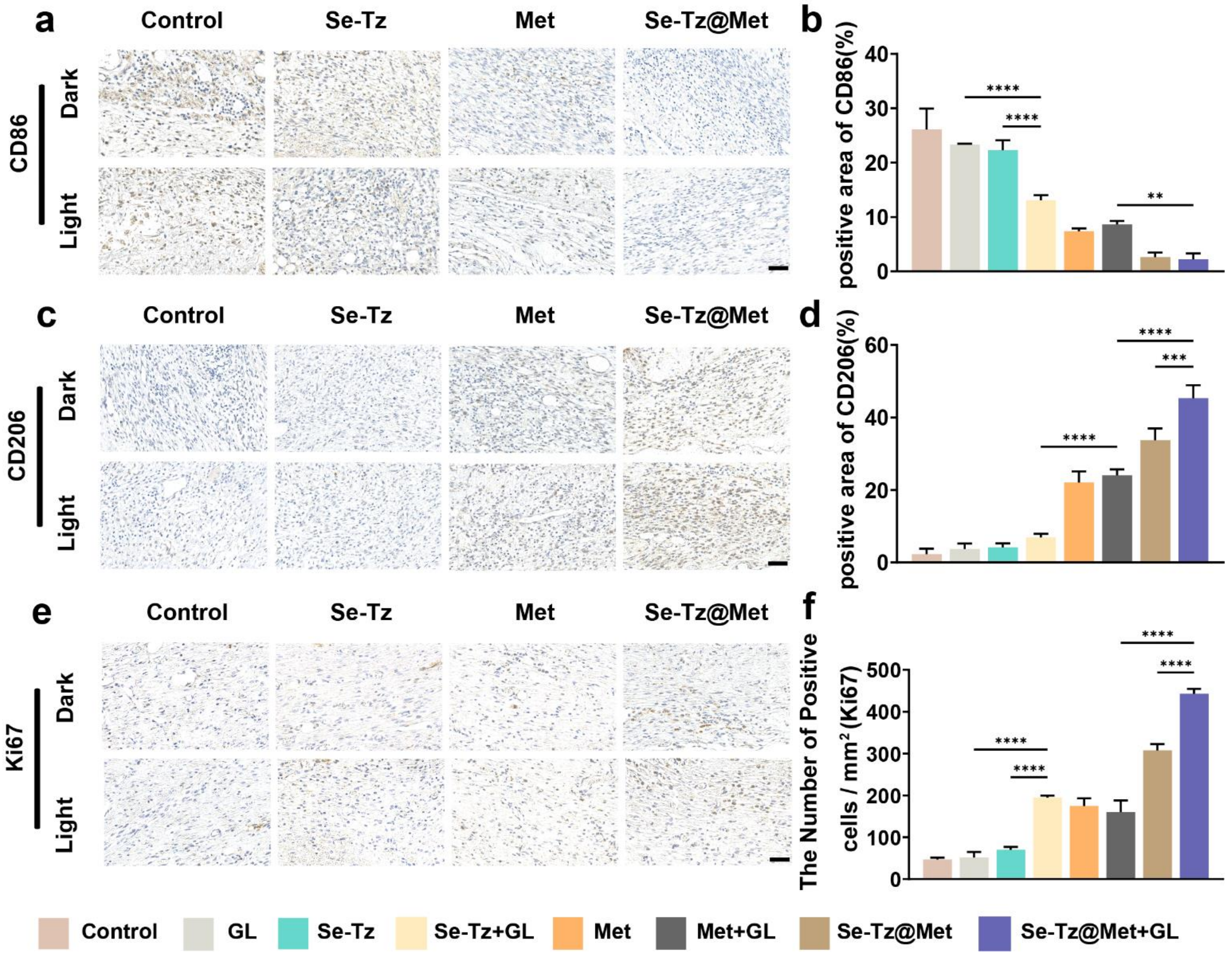

Figure S14. Histochemical staining results of CD86, CD206 and Ki67 after different treatments. (a, b) Representative images and quantification results of CD86 expression on days 6. (c, d) Representative images and quantification results of CD206 expression on days 6. (e, f) Representative images and quantification of positive stained cells of Ki67 in different groups on days 12. Scale bar: 50 μm. *P < 0.05; **P < 0.01; ***P < 0.001; ****P < 0.0001. Data are presented as the means ± SDs (n = 3).

References

[1] X. Pan, F. Driessen, X. Zhu, F. E. Du Prez, *ACS Macro Letters* **2017**, *6*, 89-92.

Author Contributions

YW: Conceptualization, Project administration, Funding acquisition. YB: Methodology, Software, Writing- Original draft preparation. WJ.L: Investigation, Visualization, Writing - Review & Editing. WH.L: Writing - Original Draft. JZ: Resources, Software. ZB.Z: Data Curation. SL: Methodology. KY.C: Formal analysis. HJ: Revised the manuscript. ML: Conceptualization, Project administration, Funding acquisition. JG: Conceptualization, Resources. XQ.P: Supervision, Project administration, Funding acquisition. All authors read and approved the fnal manuscript.
